# Treatment-associated mRNA co-expression changes in monocytes of patients with posttraumatic stress disorder

**DOI:** 10.3389/fpsyt.2023.1181321

**Published:** 2023-06-23

**Authors:** Robert Kumsta, Johannes C. S. Zang, Elisabeth M. Hummel, Svenja Müller, Dirk A. Moser, Stephan Herpertz, Henrik Kessler

**Affiliations:** ^1^Department of Genetic Psychology, Faculty of Psychology, Ruhr University Bochum, Bochum, Germany; ^2^Department of Behavioural and Cognitive Sciences, Laboratory for Stress and Gene-Environment Interplay, University of Luxemburg, Esch-sur-Alzette, Luxemburg; ^3^Department of Psychosomatic Medicine and Psychotherapy, LWL-University Hospital Ruhr-University Bochum, Bochum, Germany; ^4^Department of Psychosomatic Medicine and Psychotherapy, Fulda Hospital, University Medicine Marburg Campus Fulda, Fulda, Germany

**Keywords:** gene expression, weighted gene co-expression network analysis, posttraumatic stress disorder, monocytes, psychotherapy

## Abstract

PTSD is a prevalent mental disorder that results from exposure to extreme and stressful life events and comes at high costs for both the individual and society. Therapeutic treatment presents the best way to deal with PTSD-the mechanisms underlying change after treatment, however, remain poorly understood. While stress and immune associated gene expression changes have been associated with PTSD development, studies investigating treatment effects at the molecular level so far tended to focus on DNA methylation. Here we use gene-network analysis on whole-transcriptome RNA-Seq data isolated from CD14^+^ monocytes of female PTSD patients (*N* = 51) to study pre-treatment signatures of therapy response and therapy-related changes at the level of gene expression. Patients who exhibited significant symptom improvement after therapy showed higher baseline expression in two modules involved in inflammatory processes (including notable examples *IL1R2* and *FKBP5*) and blood coagulation. After therapy, expression of an inflammatory module was increased, and expression of a wound healing module was decreased. This supports findings reporting an association between PTSD and dysregulations of the inflammatory and the hemostatic system and mark both as potentially treatment sensitive.

## 1. Introduction

Stressful life events are associated with an increased risk for developing a range of health problems. With a life time prevalence of 1.3% to 8.8%, posttraumatic stress disorder (PTSD) is among the most common consequences directly linked to the experience of an extremely stressful event and a wide range of therapies has been developed to help patients with PTSD (1). Yet, not all people exposed to potentially traumatic incidents develop PTSD and not all patients diagnosed with PTSD benefit from psychotherapy. To tap into this diversity of responses to specific environmental conditions, several lines of research have been addressing factors associated with the etiology of PTSD or treatment response, increasingly focusing on the biological level. One goal of these efforts is to better understand biological mechanisms which are involved in the development of PTSD and mediate or predict a positive therapeutic outcome in the presence of PTSD. So far, research on etiology implicated both, the endocrine stress response as well as the immune system in PTSD development (2–4). However, many of the biological building blocks that lead to dysregulation of the immune and stress response systems and the development of PTSD are poorly understood.

Genome-wide as well as epigenome-wide studies have identified sequence variation and/or epigenetic modifications of candidate genes involved in PTSD etiology (5, 6), and recent research has begun to focus on potentially altered RNA expression in PTSD patients to gain a better understanding of the complex stress-related pathogenesis of this condition.

First gene expression studies confirmed potential effects of impaired stress processing identifying altered expression of inflammatory markers in different tissues. Gene expression analysis of patients with PTSD showed differences in the transcription of immune- and stress-associated genes compared to healthy controls (7). In addition, such changes are not limited to peripheral blood cells, but have also been found in post-mortem brains of PTSD subjects (8–10). Marchese and colleagues (11) recently found an association between gene expression and World Trade Center attack related PTSD symptom severity. Gene enrichment analyses revealed pathways related to metabolism, immune signalling, neurological signalling, and cellular structure. A meta-analysis with over 1,000 participants confirmed changes in expression of genes associated with inflammation and immune response. In a transcriptome mega-analysis, Breen and colleagues (12) analysed data from five independent PTSD studies including both genders, emergency trauma, combat trauma, assault trauma, interpersonal trauma, and childhood trauma (CT). Co-expression network analyses revealed trauma-type and gender-specific gene expression signatures. Interestingly, all investigated studies shared transcriptional changes in cytokine, innate immune, and type-1 interferon signalling pathways.

Longitudinal approaches with more than one measurement point are needed for a better understanding of the molecular changes after trauma which can lead to PTSD. Lori and colleagues (13) studied gene expression in whole blood immediately after trauma in hospitalized patients and at multiple time points after trauma. A significant difference in glutamate receptor (*GRIN3B*) and angiomotin like 1 (*AMOTL1*) gene expression was found between resilient patients and those who developed PTSD. This study design was first described by Segman et al. (14) investigating gene expression immediately after trauma and four months later in peripheral blood mononuclear cells (PBMCs). Different transcription patterns for genes involved in immune activation, regulation, proliferation, and differentiation could be found in patients developing PTSD. Studies examining gene expression before and after trauma provided insight into a causal relationship between dysregulation of genes involved in the immune system and the development of PTSD (15, 16).

These molecular signatures associated with PTSD contribute to our understanding of PTSD development and are useful tools identifying potential reporters/predictors of response to psychotherapeutic treatment. This area of molecular therapy research is still in its infancy.

Further insights into the biology of PTSD and new treatment approaches can be provided by examining patients during psychotherapeutic treatment. Changes in gene expression of genes involved in stress response pathways after successful treatment with emotional release techniques have already been shown (17). Likewise an increased expression of the FKBP prolyl isomerase 5 gene (*FKBP5*), a chaperone involved in the regulation of the stress response, after cognitive behavioral therapy was positively correlated with PTSD symptom improvement (18, 19).

Currently there is still a paucity of studies addressing the question of intervention-associated changes in gene expression in PTSD. We studied 60 female PTSD patients before and at the end of a 6 week inpatient treatment aiming to examine the mRNA expression pattern in isolated monocytes, to address potential confounds of changes in blood cell composition pre and post intervention. On the other hand, monocytes were selected because previous studies have shown that within the heterogeneous leukocyte population they appear to be the most sensitive subtype for traumatic experiences and variations in psychosocial states (20, 21). In addition changes in gene expression signature in monocytes of genes involved in inflammation as well as immune and stress responses have already been shown in patients with PTSD (22).

This study had two goals: (i) to identify pre-therapy mRNA co-expression patterns associated with subsequent treatment response and (ii) to characterize gene co-expression changes across treatment as a function of therapy response.

## 2. Materials and methods

### 2.1. Sample characteristics and questionnaires

Sixty female inpatients of European descent seeking treatment for PTSD at the Department of Psychosomatic Medicine and Psychotherapy, LWL-University Hospital, Ruhr-Universität Bochum participated in the study. The patients were between 20 and 60 years old (*M*  = 40.0, *SD* = 11.9) and had a mean BMI of 30.1 ± 7.3 (*SD*). Patients were diagnosed with PTSD (F43.1) according to ICD-10 (23) prior to inpatient admission *via* structured clinical interviews in the outpatient department of the hospital. Furthermore, symptoms of PTSD were recorded before and after inpatient treatment with the PTSD-Check List for DSM-5 (PCL-5) (24, 25). PCL-5 total scores were calculated and added across all subdimensions with higher scores indicating greater severity (0–80 points). Comparison of baseline to post treatment total scores (T0–T1) was used as criterion for therapy response. A decrease of ≥10 points on the PCL-5 total score designated patients as responder following Weathers and colleagues (24).

To assess experience of early adversity, all participants completed the German 28-item version of the childhood trauma questionnaire (26, 27), which assesses five categories of childhood adversity: sexual, physical and emotional abuse, and physical and emotional neglect. Cut-off scores representing moderate to severe exposure were applied to determine adverse experience in each of the five CTQ categories (> 12 for emotional abuse; > 9 for physical abuse; > 7 for sexual abuse; > 14 for emotional neglect; and > 9 for physical neglect). Category specific scores were used in further analyses to determine the association between type of adversity and gene module expression. Further, CTQ mean scores were calculated as an overall measure of early adverse experiences.

Patients received standard inpatient treatment for PTSD, and mean treatment duration was 6.5 ± 1.4 (*SD*) weeks. This involved symptom specific medication administered by a psychiatrist, one session each week of individual psychotherapy, three sessions of trauma group therapy, two sessions of trauma stabilization group therapy, one session of a “skills group,” two sessions of kinesitherapy, two sessions of art therapy, physiotherapy, clinical rounds, and daily short sessions with a nurse. During individual psychotherapy sessions, patients received different trauma exposure methods. The study was approved by the ethics committee of the Faculty of Psychology, Ruhr University Bochum (Nr. 155) and by the ethics committee for the Faculty of Medicine, Ruhr-University Bochum (15-5462).

### 2.2. Monocyte isolation, mRNA extraction and mRNA quantification

Nine milliliter of whole blood was drawn from each patient in the morning between 8 am and 9 am (S-Monovette 9 ml K3E, Sarstedt, Nümbrecht, Germany). Monocytes were immunomagnetically purified from whole blood with the MACS System (Miltenyi Biotec, Berglisch Gladbach, Germany), shock frozen and stored at −80°C. The homogeneity of the cells was determined with the BD FACSCanto TM II Flow Cytometer (BD Biosciences, San Jose, CA, United States), and showed high purity (*M*  = 98.3%, *SD* = 2.4%). RNA was isolated with the Maxwell 16 LEV simplyRNA Cells Kit (Promega, Fitchburg, WI, United States) according to the manufacturing protocol and stored at −80°C. Integrity of RNA was verified using the Bioanalyzer 2100 system (Agilent, Santa Clara, United States), with samples showing an average RNA integrity number (RIN) of 9.6 (*SD* = 0.5). A total of 100 ng RNA was used for genome-wide transcriptional profiling by 3´-mRNA sequencing. PolyA-selected libraries were constructed using QuantSeq 30mRNA-Seq Library Prep Kit FWD for Illumina (Cat. No. 015.24, Lexogen, Vienna, Austria) and external multiplexing barcodes for Illumina (i7 index primers 7001-7096; Lexogen, Vienna, Austria) with 12× PCR cycles for library amplification, according to manufacturer’s protocol. The fragment size and quality of the libraries were assessed by Fragment Analyzer (Advanced Analytical Technologies, Heidelberg, Germany) and sequenced with 50 bp single-end reads on a single lane of an Illumina HiSeq 2500 at the NGS Core Facility, Institute of Human Genetics, Life & Brain Research Centre, University Hospital Bonn, Germany. The mRNA sequencing data are available in the European Nucleotide Archive (ENA) under the accession number PRJEB61112.

### 2.3. Statistical analyses

#### 2.3.1. Pre-processing

Data preprocessing was performed following the workflow by Law and colleagues (28) using the R packages edgeR (29) and limma (30). Transcripts that fell below the threshold of 7 counts per million scores (CPM) or were expressed in less than 24 samples were excluded from the further downstream analyses. Data was normalized using the trimmed mean of M-values (TMM) method (31), and variance stabilizing transformation was performed using variance modeling at the observational level (voom) as implemented in the limma package (Figure A1). Seven participants with missing values on the PCL-5 or CTQ were excluded from further analyses. Sample outliers were identified based on expression data using hierarchical cluster analysis with euclidian distance and removed from the dataset (Figure A2). This resulted in a set of 14,803 transcripts in 51 patients. Ensembl identifiers of all transcripts were translated into gene symbols and the respective biotype (e.g., protein coding RNA, miRNA) by means of the Ensembl based annotation package (32) using the *Homo sapiens* reference genome GRCh38 (hg38, Ensembl version 0.86).

#### 2.3.2. Weighted gene co-expression network analysis

WGCNA was conducted using the R WGCNA package (33). The analysis was performed separately for the baseline (T0), and the joint data set (T0 and T1). This allowed to identify gene co-expression structures of potential predictive value for therapy success (baseline network, T0) and to gain insight into the stability of gene co-expression patterns across time (joint network, T0 to T1).

The joint network was constructed by combining T0 and T1 gene expression signals in a joint network approach based on the assumption that most co-expression signatures remain stability within in a confined cell type. Modules identified in this joint consensus network allow to investigate the influence of PTSD treatment intervention on module expression directly.

The two networks are based on the same sample of *N* = 51 patients and contained expression values of the same set of transcripts. To achieve a tradeoff between noise reduction, computational time, and biological meaningfulness, all transcripts remaining after preprocessing were ranked according to maximal expression variation across all samples using median absolute deviation (MAD). WGCNA was conducted on the 5,000 most differentially expressed transcripts.

First, an adjacency matrix was calculated using Pearson’s correlations between expression levels of all 5,000 genes in the datasets. Subsequently, to reach a scale-free topology index (*R*^2^) of at least 0.80, baseline matrix was raised by a power (*β*) of 8 (resulting *R*^2^ = 0.86) and the consensus matrix was raised by a power of 7 (resulting *R*^2^ = 0.89) as suggested by the pickSoftThreshold in package *function*. The adjacency matrix was further used to build up the topological overlap measure (TOM), which reflects the degree of shared neighbours between pairs of transcripts. A gene dendrogram was constructed based on TOM and modules were detected and assigned to colours using the DynamicTreeCut algorithm (34) with a minimum cluster size of 25 transcripts. To determine a reasonable number of clusters, different deep split choices were compared. A deep split of 1 was used in the baseline network and a deep split of 2 in the consensus network to generate a comparable number of larger modules across all datasets. For each module, eigengenes were calculated (MEs). MEs represent the first principal component and most of the variance of all genes comprised within the respective module. Modules with highly correlated MEs (*r* > 0.75) were merged. MEs were further used to identify modules of interest for PTSD and psychotherapeutic intervention and to define hub genes later. For the purpose of identifying changes in module expression between pre- and post-intervention, module eigengenes were retrieved for each time point, which express the extent to which the respective module is expressed at T0 or T1.

#### 2.3.3. Identifying modules of interest

Modules associated with therapy response or distinct clinical variables were considered of interest and identified by a combination of differential and correlational analyses. First, linear models were used to test whether (a) PCL-5 responders showed different ME expressions compared to non-responders in baseline measurement and (b) whether psychotherapeutic intervention had an effect on ME expression of modules identified in the joint dataset. Second, Pearson’s correlation was calculated between MEs and clinical variables as an indicator of module-trait relationships. Third, as indicator of module significance, mean absolute correlations between transcripts and clinical variables were computed. The variables considered include participants’ age and BMI as well as PCL-5 and CTQ scores. Variables reflecting dichotomous (0 = non-responder, 1 = responder) or dimensional (delta values T0–T1) therapy responses based on PCL-5 scores were included as well.

Subsequently, linear mixed effect models were fitted to modules using the R package *variancePartition* (35), to characterize the extent to which clinical variables independently explain variance in a module’s or a gene’s expression. Covariates controlled for in the different models include participants response status (PCL_r), BMI, Age, participants’ baseline score on the PCL-5 scale as well as participants’ scores on different CTQ subscales. Models used to estimate variance explained through treatment *per se* (repeated measures) include an intervention term (Int: T0/T1) modeled as a random effect.

All modules were examined to determine whether they contain differently expressed genes or genes previously reported as PTSD or trauma-related. Identification of trauma associated genes (TAGs) was based on findings reported in eight studies that investigated differential gene expression in the context of trauma and PTSD (12, 14, 21, 36–40) and the relevant data was collected from the respective articles or publicly available data repositories.

#### 2.3.4. Enrichment analysis

All identified modules were tested for gene enrichment using in-package *ImmunePathwayLists*, *brainLists*, *BloodAtlases*, and CHDI lists with the WGCNA user list enrichment function, which uses a hypergeometric test to assess gene enrichment. Further gene-ontology (GO) enrichment analysis was performed for modules of interest utilizing Enrichr (41) and adjusted Fishers exact test. To build up protein–protein interaction networks, ensembl identifiers were converted to UniProt accessions and supplied to the STRING database (42) with a combined STRING score of 40.4 (i.e., medium-to-high confidence interactions) as confidence parameter. If not mentioned otherwise, further description of distinct gene characteristics are derived from the UniProt/SwissProt database (43) and the Gene Cards suit (44).

#### 2.3.5. Identification of hub genes

Hub genes (highly connected genes) are likely to be important drivers of a modules function and were defined based on the strength of correlation between each gene and each module’s eigengene (kME). kME values were calculated across all genes of the joint dataset and separately for the modules of pre-therapy network, and genes with the highest kME values were considered as potential hub genes. For each module, 20 genes with the highest kME values are reported. Hub genes derived from modules of interest were further investigated using the variance partitioning approach and we checked whether hub genes were differentially expressed from pre- to post-therapy in differential gene expression analysis.

#### 2.3.6. Differential gene expression analysis

To validate time-related findings derived from WGCNA analyses in a subsequent step, differential gene expression (DGE) was performed to identify genes that change in expression following treatment for PTSD, irrespective of treatment response. Although *limma* is the current gold standard in gene-expression analysis, it is not particularly suited to deal with repeated measurement designs. Thus, DGE analyses were conducted using the DREAM workflow (45), which allows to account repeated measures through random effects. DREAM analysis of data was performed on filtered and normalized data (*n* = 14,803 transcripts) additionally transformed with precision weights computed on basis of fitted mixed linear models. Differential analyses were run on a minimal model including treatment (T0 to T1) as a predictor. Significance levels were set to 5% to account for multiple testing and only *p*-values that survived correction for false discovery rate (FDR) were considered as differentially expressed. Further, a cut off for meaningful fold changes was set to FC = 1.2. We report on the application of dimensionality reduction methods (hierarchical cluster analysis (HCA) and principal component analysis (PCA)) as well as on gene covariation analysis in the supplements (Figures A2–5). All analyses were carried in the R statistical environment (46).

## 3. Results

### 3.1. Sample descriptive

Due to missing questionnaire data (PCL-5 and/or CTQ), the final PTSD cohort comprised 51 patients with a mean age of 39.18 years (*SD* = 11.93, range = 20 to 60 years) and a mean BMI of 30.05 (*SD* = 7.4, range = 17.8 to 45.5). 32 participants (63%) were characterized as therapy responders based on differences between post- and pre-treatment PCL-5 scores of at least 10 points (24). The responders showed a mean difference of −24.25 ± 10.56 points, while the non-responders (*n* = 19, 37%) showed a mean difference of −0.45 ± 7.92.

Participants CTQ total score ranged from 24 to 109 (*M* = 70.25, *SD* = 19.86). 76% of all participants fulfilled CTQ cut-off criteria in more than two and 63% in more than three of the CTQ categories. Most participants received psychotropic medication (*n* = 47, 92.2%) such as antidepressants (*n* = 42, 82.4%), antipsychotics (*n* = 30, 58.8%), anxiolytics or sedatives (*n* = 10, 19.6%). Comparison by *t*-tests and Fisher–Yates-tests did not indicate significant differences between responders and non-responders in any of the variables of interest (Table 1).

**Table 1:** Sample characteristics.

	PCL-5 response			Total
	Responders	Non-responders	*p*	
Sample *n* (%)	32 (63)	19 (37)	–	51
Age mean ± *SD*	40.38 ± 12.16	37.16 ± 11.58	0.35	39.18 ± 11.93
Body mass index (BMI) mean ± *SD*	30.03 ± 7.34	30.07 ± 7.17	0.98	30.05 ± 7.40
Psychotropic medication *n* (%)	30 (93.8)	17 (89.5)	0.62	47 (92.2)
Antidepressants *n* (%)	26 (81.3)	16 (84.2)	1.00	42 (82.4)
Anxiolytics or sedatives *n* (%)	7 (21.9)	3 (15.8)	0.73	10 (19.6)
Antipsychotics *n* (%)	18 (56.3)	12 (63.2)	0.77	30 (58.8)
Anticonvulsants *n* (%)	4 (12.5)	2 (10.5)	1.00	6 (11.8)
Lithium *n* (%)	0 (0)	2 (10.5)	0.13	2 (3.9)
CTQ total score mean ± *SD*	70.85 ± 20.87	69.25 ± 18.54	0.78	70.25 ± 19.86
CTQ categories mean ± *SD*				
Sexual abuse (sa)	12.41 ± 5.42	13.32 ± 5.64	0.57	12.75 ± 5.47
Physical abuse (pa)	10.95 ± 5.48	12.00 ± 6.16	0.52	11.34 ± 5.70
Emotional abuse (ea)	16.97 ± 5.83	17.05 ± 4.57	0.95	17.00 ± 5.35
Emotional neglect (en)	18.44 ± 5.12	15.72 ± 5.67	0.08	17.43 ± 5.44
Physical neglect (pn)	12.09 ± 4.40	11.16 ± 4.98	0.48	11.75 ± 4.60
PCL-5 mean ± *SD*				
Baseline	56.62 ± 11.40	54.13 ± 12.07	0.46	55.70 ± 11.60
Post therapy	32.38 ± 14.15	53.68 ± 13.59	< 0.001	40.31 ± 17.29
Delta	−24.25 ± 10.56	−0.45 ± 7.92	< 0.001	−15.38 ± 15.06

### 3.2. Weighted gene co-expression network analysis

#### 3.2.1. Pre-therapy network

**Figure 1:**
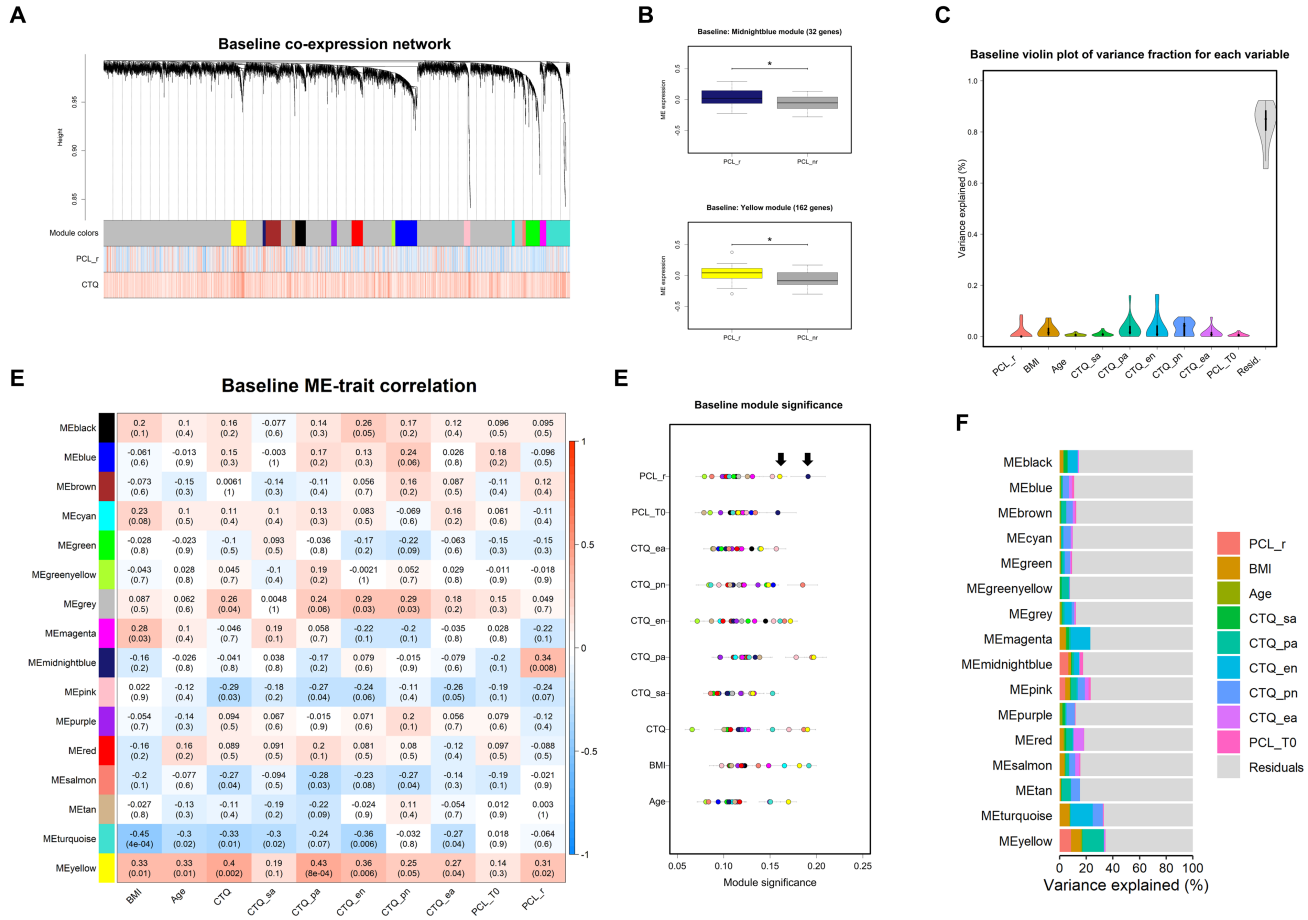
Baseline co-expression network analysis. Dendrogram **(A)** of baseline gene expression. Each line represents a transcript (leaf) and each low-hanging cluster represents a group of transcripts with similar network connections (branch) on the tree. The first band underneath the tree indicates the sixteen detected modules and subsequent bands indicate transcript-trait correlations. Red indicates a strong positive relationship and blue indicates a strong negative relationship. Note that the grey module represents a default and comprises all transcripts not assigned to another module. **(B)** Differences in module eigengene (ME) expression values between responders (colored boxes) and non-responders (grey boxes). Error bars indicates SEM and * indicates *p* < 0.05. **(C)** Violin plots represent the overall distribution of variance explained by each variable across all modules. **(D)** ME correlation with clinical variables are depicted by means of a heatmap. Blue colors indicate negative and red colors positive correlations. **(E)** Module significance for each variable. Points represent the mean absolute correlation between each transcript within a distinct module (colors) and the respective variable. Arrows point to the most significant modules: the midnight blue and yellow module showed-together with the pink module-the highest module significance scores for therapy response status. **(F)** Stacked bar plots depict partitions of variation explained by variables within each module. BMI, body mass index; CTQ, childhood trauma questionnaire (CTQ categories: sa, sexual abuse; pa, physical abuse; ea., emotional abuse; en, emotional neglect; pn, physical abuse). PCL-5, PTSD-check list for DSM-5; r, responder, T0, baseline measurement.

WGCNA identified 16 modules in the pre-therapy gene co-expression network harboring between 32 and 252 transcripts (Figure 1A and Table A1). Of all transcripts included in the analysis, 3,426 were not assigned to a specific module and are thus part of grey default module, which collects uncorrelated genes. Overall distribution of variance explained by each variable across all modules is depicted in Figure 1C.

We first addressed the question whether pre-treatment co-expression patterns would be predictive of therapy outcome. The module eigengenes of the midnight blue (*r* = 0.34, *p* < 0.01) and the yellow (*r* = 0.31, *p* < 0.05) module were positively correlated with PCL-5 response status (Figure 1B) and the midnight blue and yellow module showed-together with the pink module-the highest module significance scores for therapy response according to the PCL-5 criterion (Figures 1D–F). Linear models showed that therapy responders had significantly stronger expression before treatment of the midnight blue (32 genes) *β* = 0.09*, p* < 0.05, *F* (1, 47) = 6.59, *p* < 0.05, *R^2^* = 0.10, and the yellow (162 genes) module *β* = 0.09*, p* < 0.05, *F* (1, 47) = 5.30, *p* < 0.05, *R^2^* = 0.07 compared to non-responders.

**Figure 2:**
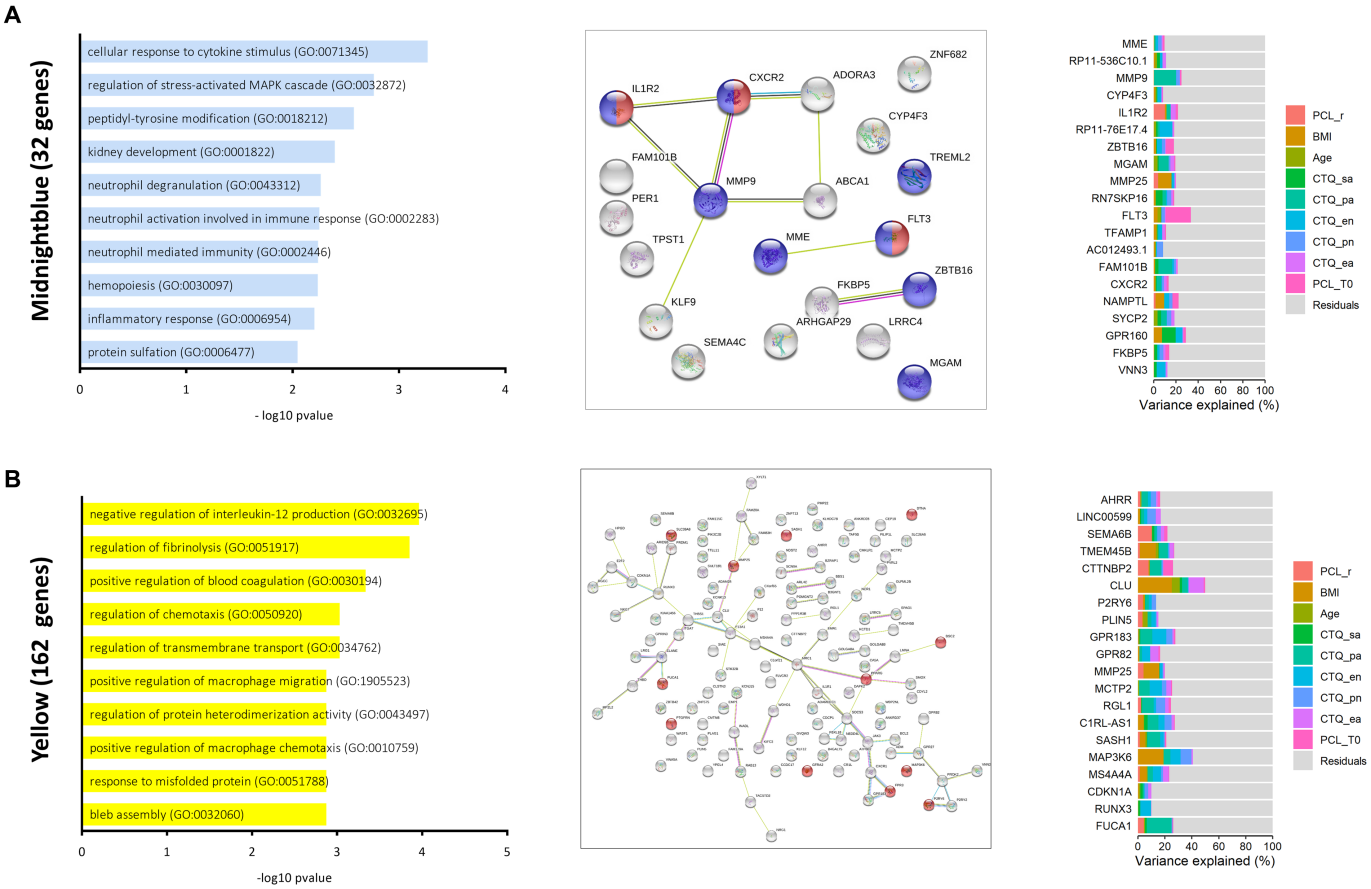
Modules of interest in the baseline gene co-expression network. Bar plots (left) show the top ten most significantly enriched biological processes ranked by-log10 *p*-values for the midnight blue **(A)** and the yellow **(B)**. String networks were generated for each module (middle). Colours indicate functional associations within the midnight blue module (red = cytokine receptor activity, blue = immune system), the yellow module [red = genes referenced by Verdugo et al. (48)]. The connecting lines indicate different evidence levels used by the STRING database to describe the nature of the displayed connection between to proteins. Known Interactions: blue = from curated databases, pink = experimentally determined, Predicted Interactions: green = gene neighborhood, red = gene fusions, blue = gene co-occurrence, others = yellow = examining, black = co-expression, lightblue = protein homology. Stacked bar plots (right) report fractions of variation explained by a set of variables within the top 20 hub proteins of reported modules.

After controlling for all other variables, therapy response status explained 6.6% of variance of the midnight blue module’s expression. The midnight blue module contains transcripts, among others, functionally implicated in inflammatory response (GO: 006954), cellular response to cytokine stimulus (GO: 0071345) and regulation of adenylate cyclase activity (GO: 00077190) (Figure 2A). Of all genes assigned to the module (*n* = 32), four were found differentially expressed after psychotherapeutic intervention in DGE analyses (*IL1R2*, *ABCA1*, *KLF9* and *LRRC4*) and seven were previously reported as trauma associated (*MME*, *TREML2*, *MMP9*, *IL1R2*, *ABCA1*, *NAMPTP1* and *FKBP5*, a major regulator of the glucocorticoid mediated stress response, and previously associated with PTSD (49)).

The 20 genes with the highest conserved kME values are reported in Table A2 and the top five hub genes of the midnight blue module include *MME*, *RP11-536C10.1*, *MMP9*, *CZP4F3* and *IL1R2*. Variance partitioning of these hub genes indicated that especially *IL1R2* (10.0% of explained variation), *TFAMP1* (6.35% of explained variation) and *NAMPTL* (1.54% of explained variation) drive the modules association with therapy response and all three might present potential candidates in search of predictive markers of therapy success (Figure 2A).

For the other module of interest (yellow), therapy response explained 8.5% of variance in expression values and variance portioning further indicated an association between different forms of early adverse experience and expression of the yellow module. The yellow module (Figure 2B) was enriched for transcripts functionally involved in negative regulation of interleukin 12 production (GO: 0032695), regulation of fibrinolysis (GO: 0051917) and positive regulation of blood coagulation (GO: 0030194). Of all genes assigned to the yellow module (*n* = 162), 14 were differentially expressed after treatment and 44 were previously reported as trauma associated.

Across all 20 genes with the highest kME values (about 12% of transcripts in the yellow module) PCL-5 response explained up to 10% of variance (*M* = 2.0%, *SD* = 2.8%). BMI explained a mean variation of 4.4% (*SD* = 0.7%) of module expression, and CTQ physical abuse scores explained a mean variation of 5.34% (*SD* = 4.36%) of variance across all 20 hub genes.

The top 5 hub genes of the yellow module (see Tables A2, A3 for the 20 genes and their biotypes with the highest kME values in ranked order) included *AHRR*, *LINC00599*, *SEMA6B*, *TMEM45B* and *CTTNBP2*. Of note, *AHRR* was previously implicated in a large PTSD epigenome-wide association study (6). Variance partitioning revealed that therapy response explained mainly variation in expression of *SEMA6B* (10.1%), a protein coding gene of the semaphorin family reported to be involved in monocyte-endothelium adhesion and transmigration (47, 50) and in expression of *CTTNPP2* (7.8%), a protein involved in regulation of cytokine mediated pro-inflammatory signalling (51).

#### 3.2.2. Joint network

**Figure 3:**
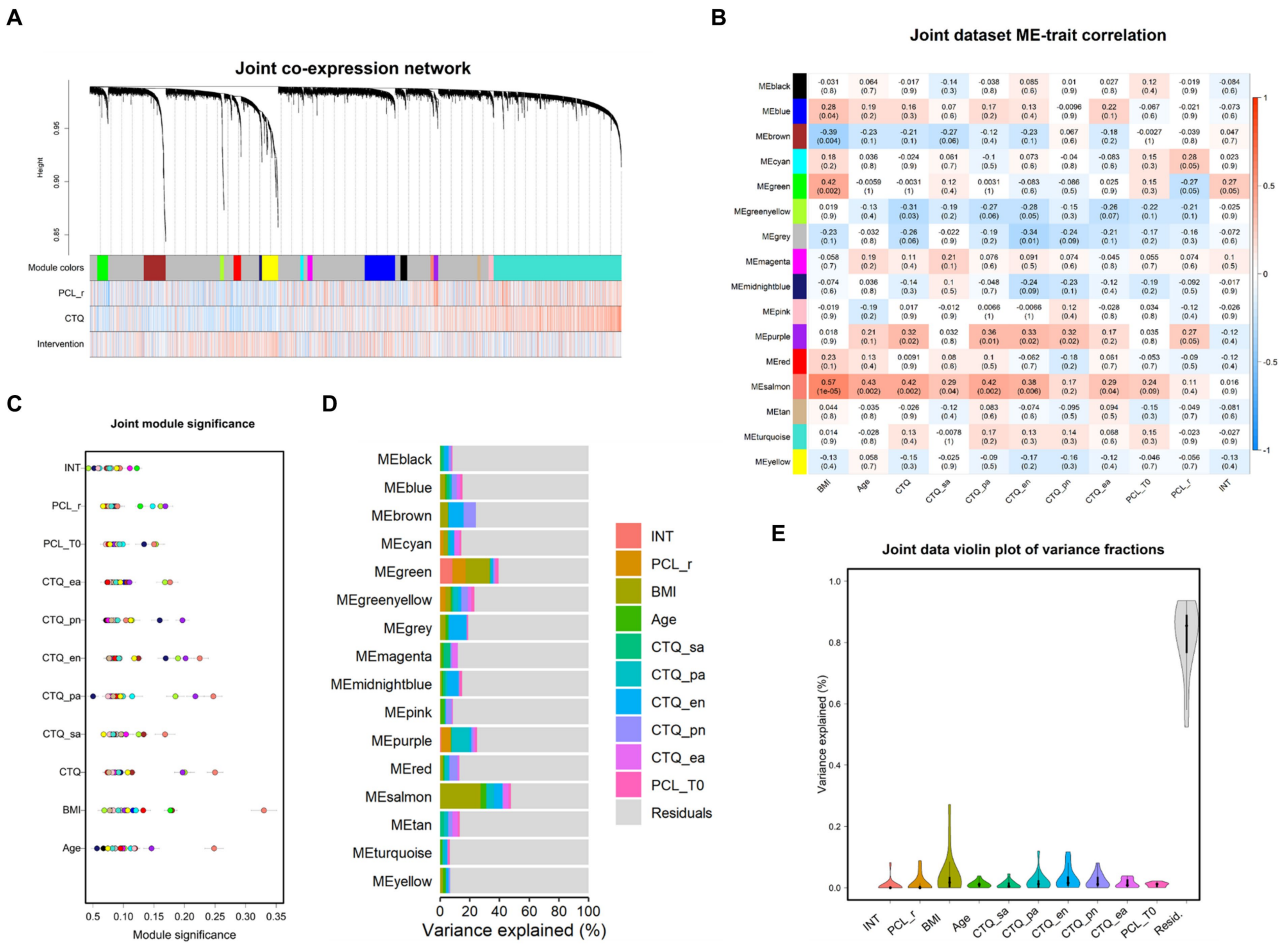
Joint data co-expression network analysis. **(A)** Dendrogram of gene expression. Each line represents a transcript (leaf) and each low-hanging cluster represents a group of transcripts with similar network connections (branch) on the tree. The first band underneath the tree indicates the fifteen detected modules and subsequent bands indicate transcript-trait correlations. Red indicates a strong positive relationship and blue indicates a strong negative relationship. Note that the grey module represents a default and comprises all transcripts not assigned to another module. **(B)** Module eigengene (ME) correlation with clinical variables are depicted by means of a heatmap. Blue colors indicate negative and red colors positive correlations. Module significance for each variable is depicted in **(C)**. Points represent the mean absolute correlation between each transcript within a distinct module (colors) and the respective variable. **(D)** Stacked bar plots depict partitions of variation explained by the displayed set of variables within each module. **(E)** Respectively, distribution of variance explained by each variable across all modules is depicted in the violin plot. BMI, body mass index; CTQ, childhood trauma questionnaire (CTQ categories: sa, sexual abuse; pa, physical abuse; ea., emotional abuse; en, emotional neglect; pn, physical neglect). PCL-5 = PTSD-check list for DSM-5; r, responder; T0, baseline measurement; T1, post therapy measurement; d, delta T1–T0; intervention = baseline or post therapy measurement.

The goal of WGCNA on the joint dataset (i.e., pre- and post-therapies expression values) was to test whether treatment is associated with changes in gene expression. In the joint-data gene co-expression network, 15 modules comprising between 26 and 1,199 genes were detected (Table A1). 2,630 of the original 5,000 transcripts were assigned to the grey default module (Figure 3A and Table A1).

**Figure 4:**
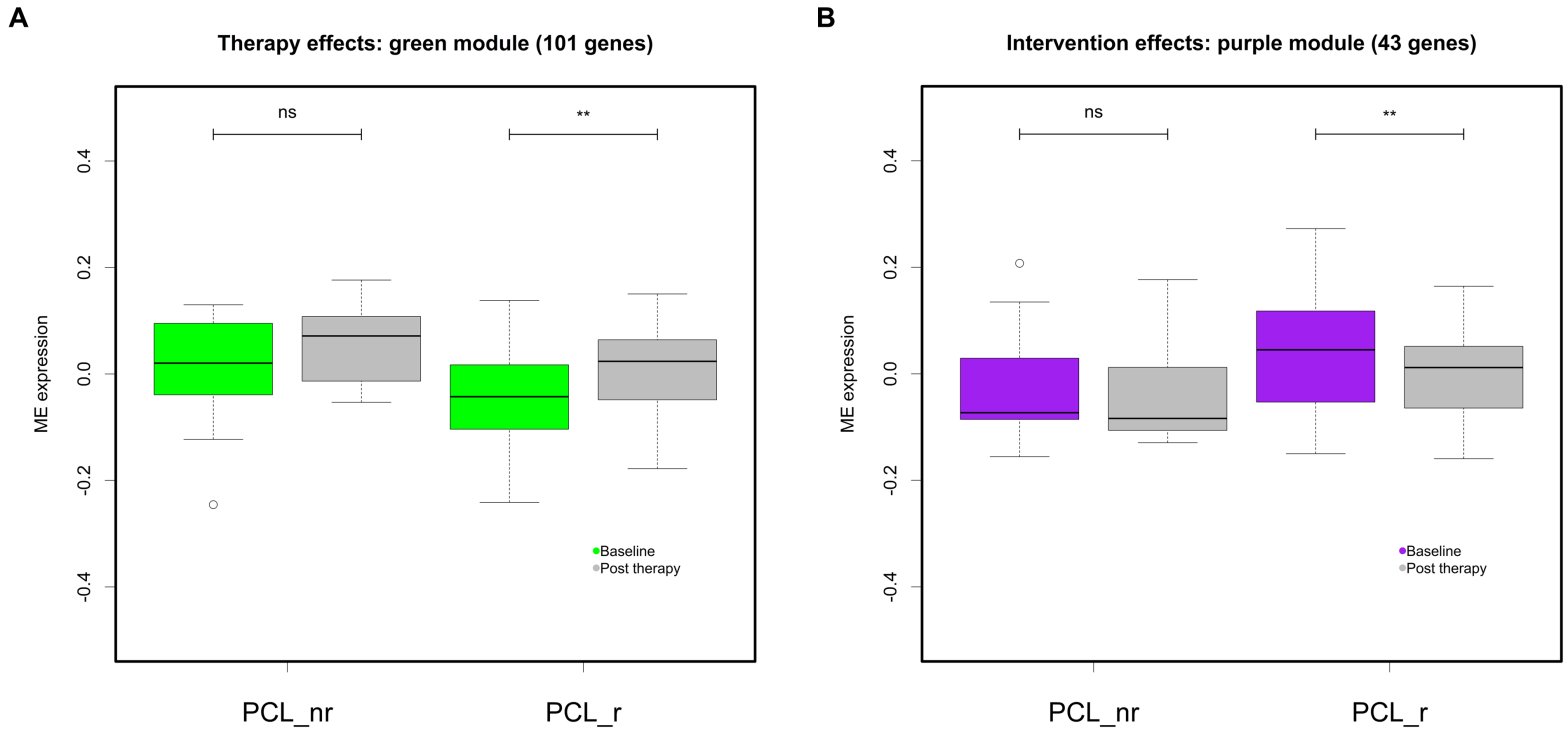
Differential module expression after therapy. Boxplots depict baseline and post therapy eigengene expression of the green **(A)** and the purple module **(B)** across all participants (all), and separately for participants characterized as responders (PCL_r) and non-responders (PCL_nr). ** = *p* < 0.01. Error bars indicate SEM.

Gene significance analyses (Figure A5) and subsequent module significance analyses (Figure 3C) indicated expression of the green module (101 genes) and the purple module (43 genes) as sensitive to treatment. The green module displayed high significance values for the PCL-5 response, but also for therapy *per se*, indicating change over time irrespective of treatment response, and BMI. Repeated-measure analysis of variance (ANOVA) revealed a significant main effect for therapy on expression values of the green module *F* (1, 49) = 15.04, *p* < 0.001, *η*_ges_ = 0.09, with *eigengene* values increasing after therapy (Figures 3B and 4A). FDR corrected post-hoc comparisons showed a significant increase in module expression of PCL-5 responders after therapy (*p* < 0.01) but not in expression values of participants characterized as PCL-5 non-responders (*p* = 0.121).

Expression of the purple module significantly decreased after treatment *F* (1, 49) = 6.46, *p* < 0.05, *η*_ges_ = 0.01. FDR corrected post-hoc *t*-test comparisons showed significant decrease after therapy in PCL-5 responders (*p* < 0.01), but not in non-responders (*p* = 0.33; Figure 4B).

Across all modules, treatment explained a maximum of variation of expression of the green module (8.16%), and therapy response status explained 8.86% variation of the green module’s expression (Figure 3D). Regarding the purple module, intervention did not account for variation in the purple module’s expression after controlling for all other variables. It is of note that participants’ BMI explained variation of 2.1%–27.1% across all modules (Figure 3E) and accounted for considerable shares of variation especially in the expression of individual modules (up to 27.1%; salmon module; Figures 3B,D). Across all modules, participants’ scores on the CTQ subdimensions explained up to 3.2% of mean variation in expression (CTQ emotional neglect: *M* = 3.15, *SD* = 0.3%), and the CTQ emotional neglect score explained most variation of the brown module (10.3%) and the midnightblue module (7.6%).

**Figure 5:**
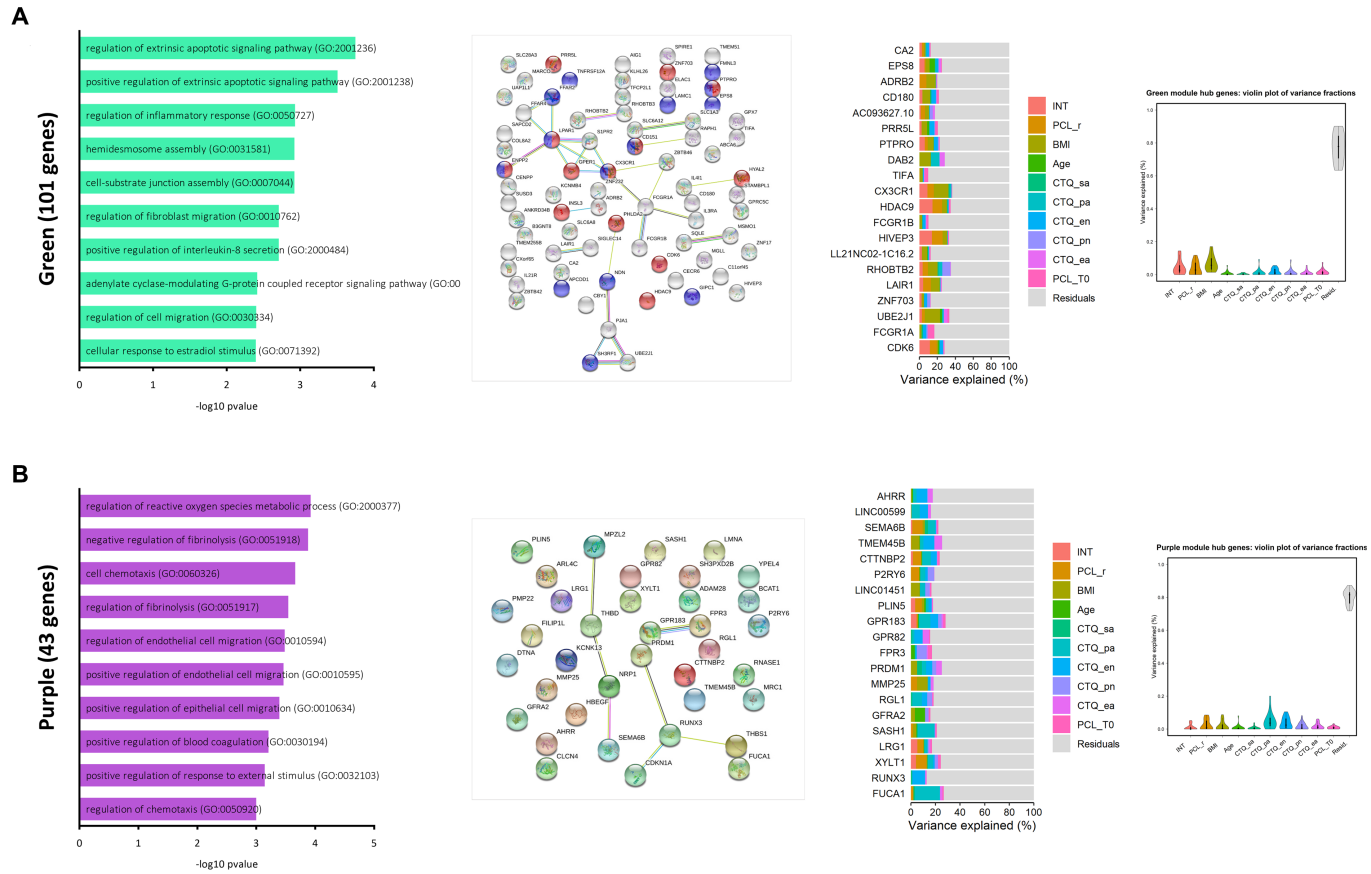
Modules of interest in the joint data gene co-expression network. Bar plots (left) show the top ten most significantly enriched biological processes ranked by-log10 *p*-values for the green **(A)** and the purple module **(B)**. String networks were generated for each module (middle). Colours indicate functional associations within the green module (red = regulation of cellular component movement, blue = cell motility). The connecting lines indicate different evidence levels used by the STRING database to describe the nature of the displayed connection between to proteins. Known Interactions: blue = from curated databases, pink = experimentally determined, predicted interactions: green = gene neighborhood, red = gene fusions, blue = gene co-occurrence, others = yellow = examining, black = co-expression, lightblue = protein homology. Stacked bar plots report partitions of variation explained by a set of variables within the top 20 hub genes of the reported module. Violin plots depict total variation explained by each variable across all 20 hub genes.

The green module harbors genes involved in regulation of apoptotic pathways (GO: 2001238, GO: 2001238) and inflammatory response (GO: 0050727). It is further enriched for genes functioning in regulation of interleukin 8 secretion (GO: 2000484) and assembly of hemidesmosomes (GO: 0031581), cell structures that are important for cellular attachment processes (Figure 5A). The module contains 16 TAGs and 7 genes that were differentially expressed after therapy. Of all 101 transcripts assigned to the module, a majority of 67% is protein coding. When supplied to the string database, these transcripts form a clearly connected protein-interaction network structure with the *C-X3-C motif chemokine receptor 1* (*CX3CR1*), the *lysophosphatidic acid receptor 1* (*LPAR1*) and the *Fc gamma receptor 1a* (*FCGR1A*) as central nodes.

Among the 20 genes with the highest intramodular connectivity values (hub genes), five were differentially expressed after treatment (*CDK6, CX3CR1, EPS8, HDAC9, HIVEP3*) and eleven were reported as TAGs in previous studies (*FCGR1B*, *CD180*, *CA2*, *UBE2J1*, *TIFA*, *LAIR1*, *FCGR1A*, *CDK6*, *PTPRO*, *PRR5L*, *ADRB2*). Therapy explained mean variation of 4.3% (*SD* = 4.5%) in expression of these genes, further variance in expression was explained by the PCL-5 response criterion (*M* = 3.7%, *SD* = 4.0%). BMI explained between 1.4%–17% of the mean expression variation (*M* = 6.9%, *SD* =4.5%) in the twenty most important intramodular genes. Therapy explained most variation in expression of *HDAC9* (14.4%), *HIVEP3* (14.0%), *CDK6* (11.7%) and *CX3CR1* (8.9%) (Figure 5A and Tables A4, A5).

The purple module contains altogether 43 genes, functionally associated with the regulation of reactive oxygen species metabolic processes (GO: 2000377), negative regulation of fibrinolysis (GO: 0051918), cell chemotaxis (GO: 0060326), and positive regulation of blood coagulation (GO: 0030194) (Figure 5B). The module contains 12 TAGs (*AHRR, LMNA, XYLT1, CDKN1A, TMEM45B, FPR3, SASH1, MMP25, THBS1, LINC01451, GPR183* and *RGL1*) and 8 genes that showed significant different expression after therapy (*SEMA6B, THBS1, AHRR, GPR183, SH3PXD2B, LMNA, LRG1* and *XYLT1*).

Among the 20 hub genes with the highest intramodular connectivity values in the purple module, five were differentially expressed after psychotherapeutic intervention (*LRG1*, *SEMA6B*, *AHRR*, *XYLT1* and *GPR183*) and 13 reported as TAGs in previous studies (*AHRR, FPR3*, *MMP25*, *FUCA1*, *SASH1*, *RUNX3*, *RGL1*, *GPR82*, *LINC01451*, *TMEM45B*, *XYLT1*, *LRG1* and *GPR183*). This marks especially *AHRR, XYLT1*, *GPR183*, and *LRG1* as genes of interest within the purple module (Figure 5B and Tables A4, A5).

### 3.3. Differential gene expression

**Figure 6:**
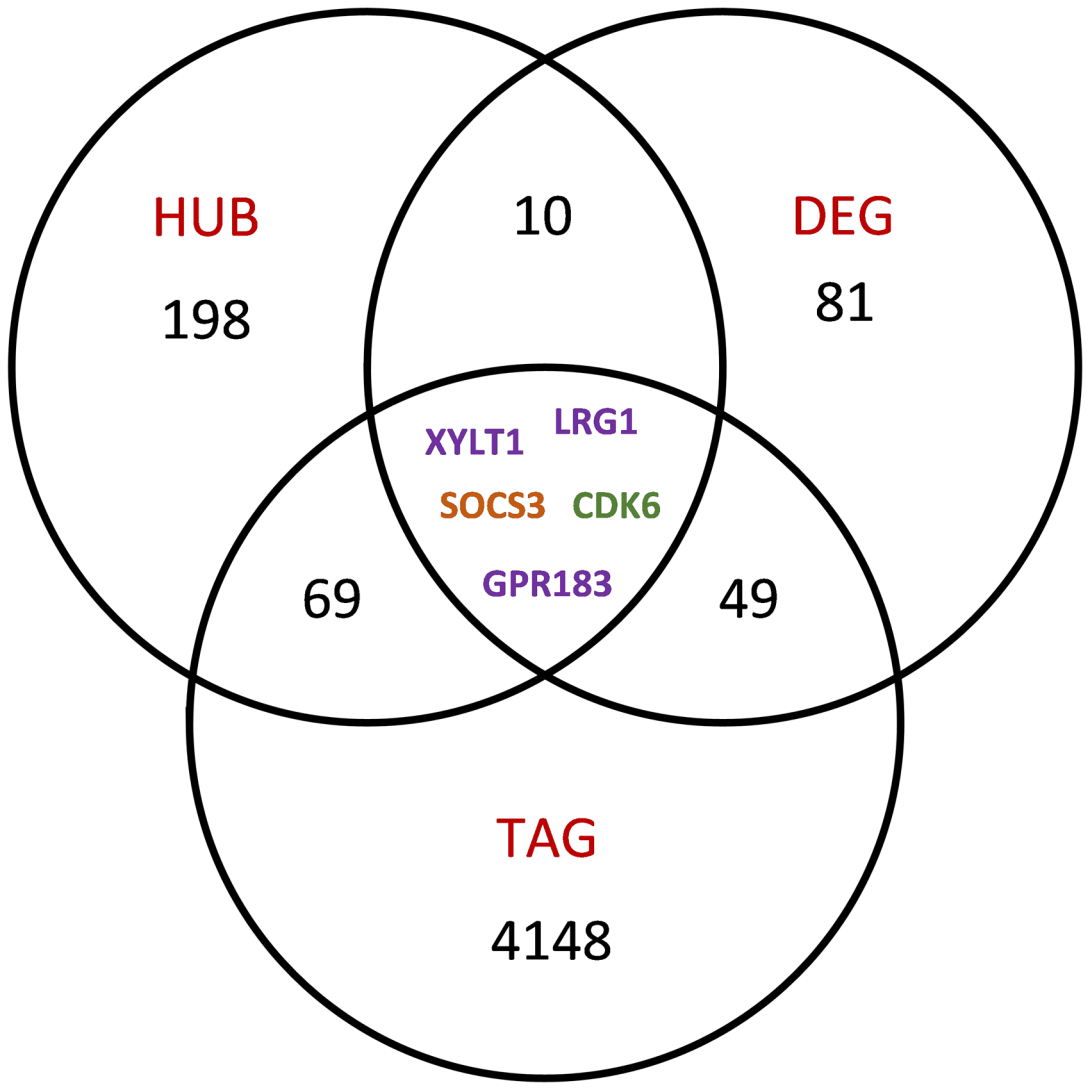
Transcripts of further interest. A Venn diagram depicts the intersection of transcripts identified as HUB genes in the joint network, as therapy response genes (DEG) and of genes reported as trauma associated within the broader literature (TAG). Transcripts are color coded according to co-expression module membership in the joint analysis network.

There were 145 transcripts that were differentially expressed from pre- to post-treatment (*p* < 0.05 FDR corrected) and had fold changes of |≥1.2|. Most transcripts (*n* = 98) were downregulated in comparison to pre-treatment measurement 0.54 (37%) of the genes identified as differentially expressed after intervention were reported as trauma associated genes (TAGs) in previous studies (Figure 6; see also Supplementary Tables A6, A7 for enrichment analysis). There was no evidence for significant expression differences between participants that responded to PTSD treatment and those that did not respond.

## 4. Discussion

We compared the RNA expression profiles of 51 PTSD patients in purified monocytes before and after 6 weeks of therapy. The two goals of the study were to identify gene co-expression signatures associated with subsequent therapy success, and to investigate changes in gene expression activity over time associated with therapy response in PTSD patients. Patients who showed a clinically significant reduction of PTSD symptoms after inpatient treatment did not differ from patients characterized as non-responder before treatment in clinical or other variables such as age, BMI, or extent of reported childhood adversity. On the molecular level, however, pre-therapy analyses indicated gene co-expression differences between patients who responded to intervention and participants who did not. Therapy responders showed significantly stronger expression of two groups of co-expressed genes, the midnight blue (32 genes) and the yellow (162 genes) module. Eigengenes of both these modules were positively correlated with PCL-5 change, and therapy status continued to explain variation in expression of both modules after controlling for confounders. Both modules contained altogether 51 genes (26%) previously associated with PTSD and experience of trauma.

The midnight blue module is functionally involved in inflammatory processes and response to cytokine stimulus. Elevated proinflammatory signaling has been consistently associated with PTSD and traumatic stress. Notable genes of the midnight blue module include *IL1R2*, an interleukin 1 family receptor. IL-1 family cytokine members activate innate inflammation and *IL1R2* was reported as elevated in PTSD candidates four months after potentially traumatic experiences. In the present study, response to therapy explained a substantial amount of variation in *IL1R2* baseline expression (6.6%) and following treatment, *IL1R2* expression was found significantly reduced. Co-expression of *IL1R2* with *FKBP5* underscores the modules’ function in inflammatory signalling. *FKBP5* is a major negative regulator of the glucocorticoid mediated stress response (52). Higher concentrations of *FKBP5* promote inflammation, and *FKBP5* has been consistently associated with PTSD-related disturbances in glucocorticoid mediated stress signaling (49).

The yellow module (Figure 2B) was enriched for transcripts functionally involved in negative regulation of interleukin 12 production (GO: 0032695), regulation of fibrinolysis (GO: 0051917) and positive regulation of blood coagulation (GO: 0030194). In previous WGCNA analyses, PTSD was associated with increased expression of modules implicated in wound healing (12, 36), like the yellow module in the present study, and this further supports the idea that coagulation processes play a role in PTSD biology.

Analysis of gene co-expression from pre-to-post therapy showed that activity of two modules changed in responders, but not in therapy non-responders. Expression of the purple module, which shares a significant number of genes (*n* = 36, 83%) with the pre-therapy yellow module (the “wound healing module”), decreased after PTSD treatment, especially in PCL-5 responders. Like the yellow module, the purple module was also enriched with genes functionally involved in negative regulation of fibrinolysis and positive regulation of blood coagulation, further implicating wound healing processes. These results suggest that processes fostering the formation of blood clots appear to be downregulated in those patients who profit from the intervention.

Expression of the green module increased after therapy. The green module is implicated in the regulation of apoptotic pathways and inflammatory processes. It contains genes involved in the regulation of interleukin 8 secretion, which is a biomarker for acute inflammation processes. Thus, whereas PTSD has been consistently associated with increased inflammatory processes, the observed expression dynamic suggests inflammation to even increase after therapy. Our results are further at odds with reports by Rusch and colleagues (39), who reported downregulated immune response system and metabolic networks with a *NF-κB* hub in PTSD affected, almost exclusively male, military service participants who showed symptom improvement after 4 to 8 weeks of evidenced-based sleep treatment.

This highlights one limitation of our study design, namely the lack of further follow-up measures. It is conceivable that increased expression of this module is part of a physiological readjustment process, and that changes in symptoms precede further downstream changes in gene co-expression and ultimately immune function. On the other hand, it needs to be noted that function inferred through gene enrichment analysis might not necessarily reflect actual physiological functioning, so caution is generally warranted when using bioinformatic inference methods.

Further limitations deserve mentioning. The study design did not include a control group, and there is no way to determine whether effects can be causally attributed to the influence of therapy or due to other influences, such as changes in cell composition within the CD14^+^ pool or factors unrelated to intervention. The sample was composed of female patients only to exclude the confounding factor sex. Gene regulation in context of PTSD has been reported to differ between females and males (38), so it remains unclear whether found results can be generalized to a male sample. Furthermore, only patients of European origin took part in the study. Unfortunately, we cannot make any statement about patients with other origins. The sample size was small for a traditional analysis on single transcript level, but adequately powered for co-expression analysis (33). Despite these limitations, the fact that therapy responders and non-responders showed differences in baseline expression and that modules of interest often contained genes previously linked to PTSD and trauma lends confidence to the findings.

Altogether, in single transcript analyses, we identified 145 genes, which were differentially expressed in participants after PTSD treatment (DEG) and 15 of these genes showed to be particularly important within their respective co-expression modules (HUB genes). Seventy-four of the joint network HUB gene candidates and 54 of identified DEGs were reported as trauma-associated before (TAGs) and 5 transcripts (*XYLT1, LRG1, SOCS3*, *CDK6* and *GPR183*) fulfilled all criteria, i.e., were identified as hub genes, were differentially expressed after intervention, and were previously reported as trauma-associated.

## 5. Conclusion

To conclude, our results lend insights into differential gene co-expression related to therapy success in PTSD patients and highlight gene expression profiles that are potentially sensitive to treatment or might be predictive of response to therapeutic intervention. Analyses support previous findings associating PTSD with dysregulation of immune system functioning and processes involved in blood coagulation and wound healing, and mark both as potentially therapy sensitive. Confirmatory studies are needed to follow up on these results, and findings of this exploratory study should be considered hypothesis generating. Future approaches could particularly benefit from larger sample sizes and the inclusion of a control sample to causally link gene expression changes to intervention. In addition, the realization of more sampling points and a post-therapy follow-up measurement could contribute to a better understanding of changes in gene expression dynamics during therapy. To better understand the molecular mechanics that may mediate psychotherapeutic effects, it would be ideal to integrate the study of gene expression with DNA methylation analysis and proteomics while measuring endocrine correlates of stress and arousal.

## Data availability statement

The original contributions presented in the study are publicly available. This data can be found here: European Nucleotide Archive (ENA) under the accession number PRJEB61112, link: https://www.ebi.ac.uk/ena/browser/view/PRJEB61112.

## Ethics statement

The studies involving human participants were reviewed and approved by the Faculty of Psychology, Ruhr University Bochum (Nr. 155) and by the ethics committee for the Faculty of Medicine, Ruhr-University Bochum (15-5462). The patients/participants provided their written informed consent to participate in this study.

## Author contributions

RK, DM, and HK developed the study concept. RK, HK, and SH contributed to the project administration. SH and HK recruited the patients. DM, EH, and SM developed the methods and prepared the samples for analysis (and run all wet lab analyses). JZ and RK developed the statistical analysis. JZ, RK, and EH conducted the statistical analyses. JZ, EH, and RK wrote a first manuscript draft. RK, HK, JZ, EH, and DM were involved in finalising the manuscript. All authors contributed to the article and approved the submitted version.

## Funding

This work was supported by a project grant by the German Research Foundation (DFG) to RK (KU 2479/3-1, KU 2479/3-2).

## Conflict of interest

The authors declare that the research was conducted in the absence of any commercial or financial relationships that could be construed as a potential conflict of interest.

## Publisher’s note

All claims expressed in this article are solely those of the authors and do not necessarily represent those of their affiliated organizations, or those of the publisher, the editors and the reviewers. Any product that may be evaluated in this article, or claim that may be made by its manufacturer, is not guaranteed or endorsed by the publisher.
