# Supplementary material for: Treatment-associated mRNA co-expression changes in monocytes of patients with posttraumatic stress disorder
Source: Front Psychiatry. 2023 Jun 23;14:1181321. doi: 10.3389/fpsyt.2023.1181321 (PMC10326517; doi:10.3389/fpsyt.2023.1181321)
Supplement: Supplementary file 1 [file Data_Sheet_1.PDF]

Appendix A. Supplementary Information

This section includes the following complementary figures

FIGURE A.1: EFFECTS OF PREPROCESSING STEPS ON DATA QUALITY. 2

FIGURE A.2: OUTLIER DETECTION BASED ON HIRACHICAL CLUSTERING WITH EUCLIDIAN DISTANCY. 3

FIGURE A.3: PCA PLOTS FOR JOINT BASLEINE AND POST THERAPY GENE EXPRESSION VALUES. 4

FIGURE A.4: BASELINE AND POST THERAPY GENE SIGNIFICANCE 5

FIGURE A.5: JOINT DATA GENE SIGNIFICANCE 6

This section includes the following complementary tables

TABLE A.1: MODULE SIZE AND OVERLAPP WITH DIFFERENTIALLY EXPRESSED GENES AND TRAUMA ASSOCIATED GENES FOR ALL CO-EXPRESSION NETWORKS 7

TABLE A.2: TOP 20 TRANSCRIPTS WITH HIGHEST KME VALUES FOR ALL BASELINE MODULES. 8

TABLE A.3: BIOTYPES OF TOP 20 TRANSCRIPTS WITH HIGHEST KME VALUES FOR ALL BASELINE MODULES. 9

TABLE A.4: TOP 20 TRANSCRIPTS WITH HIGHEST KME VALUES FOR ALL MODULES OF THE JOINT DATA CO-EXPRESSION NETWORK. 10

TABLE A.5: BIOTYPES OF THE TOP 20 TRANSCRIPTS WITH HIGHEST KME VALUES FOR ALL MODULES OF THE JOINT DATA CO-EXPRESSION NETWORK. 11

TABLE A.6: ENRICHED GO-TERMS FOR GENES UPREGULATED FOLLOWING THERAPY. 12

TABLE A.7: ENRICHED GO-TERMS FOR GENES DOWNREGULATED FOLLOWING THERAPY. 13

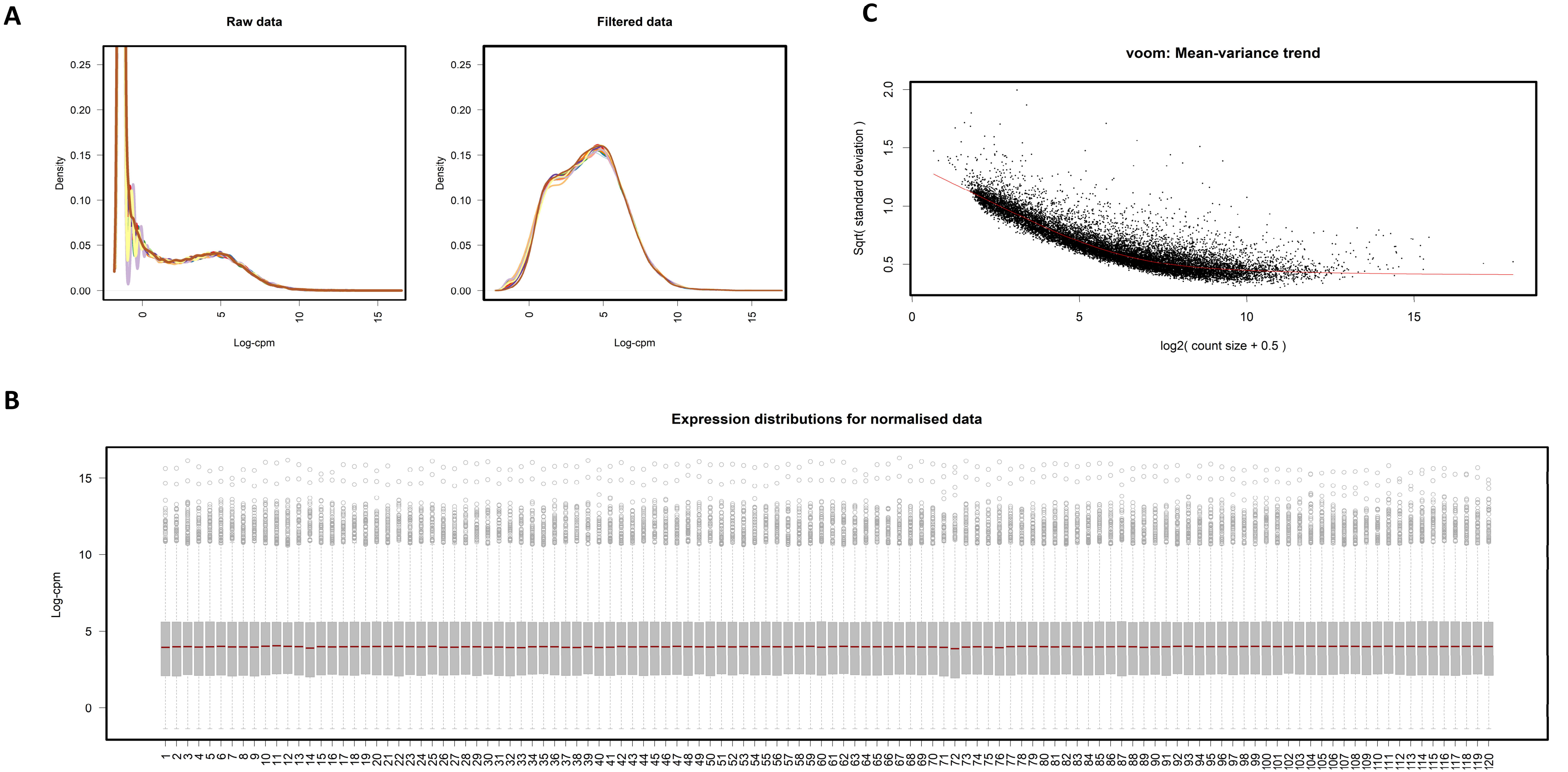

Figure A.1: Effects of preprocessing steps on data quality.

The density of log-CPM values is given for raw data and filtered data (A). Boxplots of log-CPM values visualize the expression distributions for TMM normalized data. Red bars indicate medians (B). A voom plot (c) shows the dependency of log2 mean values and the square-root residual variance of each transcript in normalized data before transformation. The decreasing trend between means and variances indicates sufficient filtering.

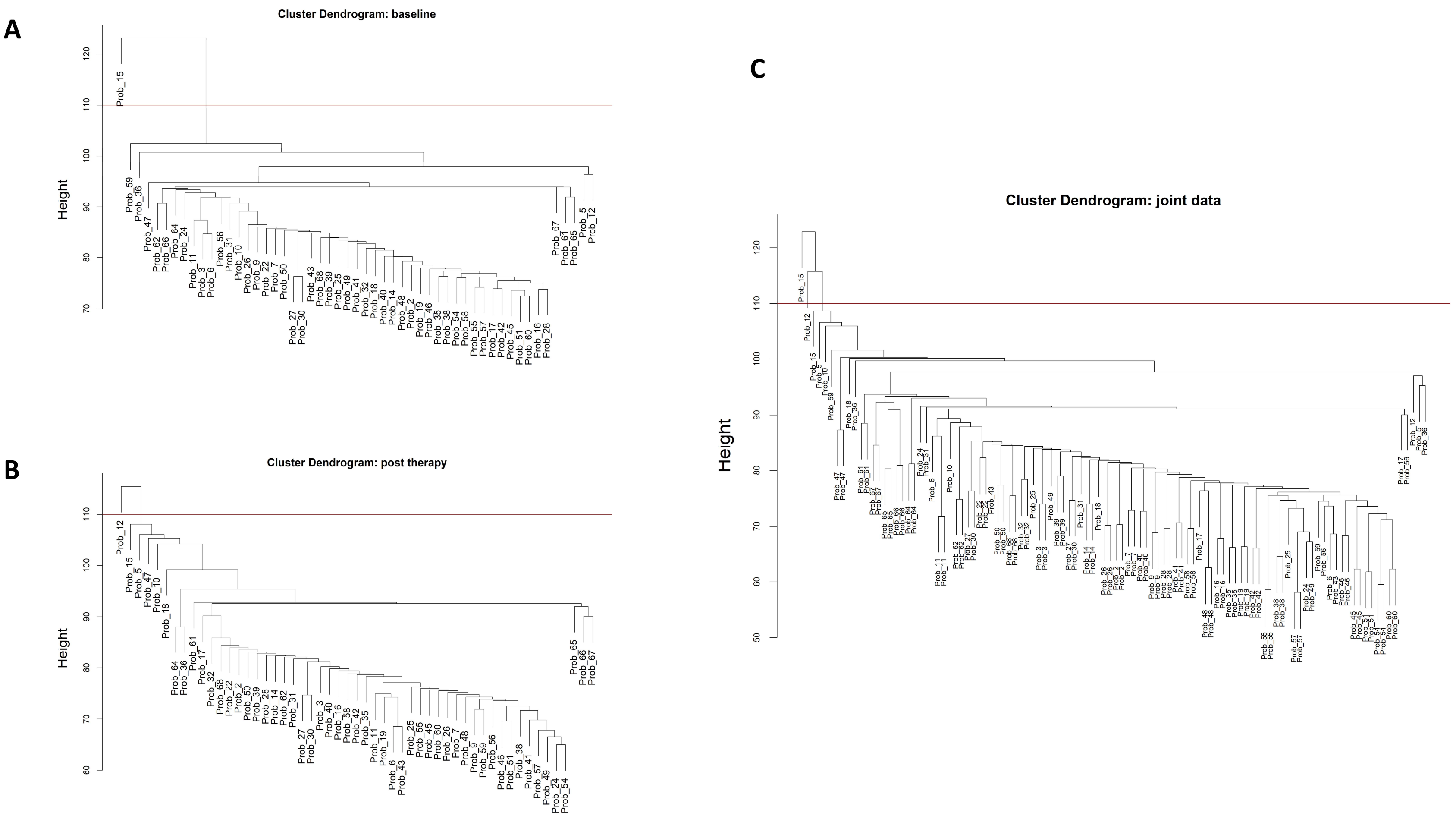

Figure A.2: Outlier detection based on hierarchical clustering with Euclidian distance.

Dendrograms for the baseline (A), post therapy (B) and the joint network (C) are depicted. Outliers above the cutoff (red line) were removed from the further analysis.

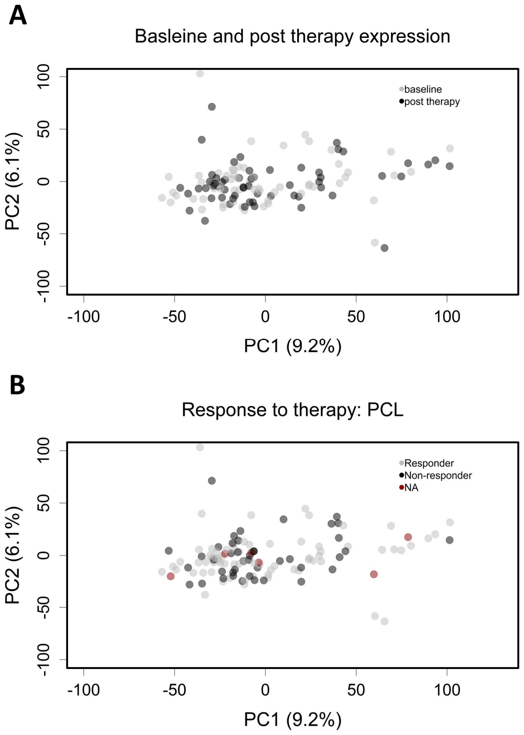

Figure A.3: PCA plots for joint baseline and post therapy gene expression values.

The first PC accounted for 9.2 % and the second PC for 6.1 % of variation in gene expression. Participant's values on the first and second extracted PC are depicted. Points characterize baseline and post therapy measurement (A) and therapy response according to the PCL-5 (B) criteria.

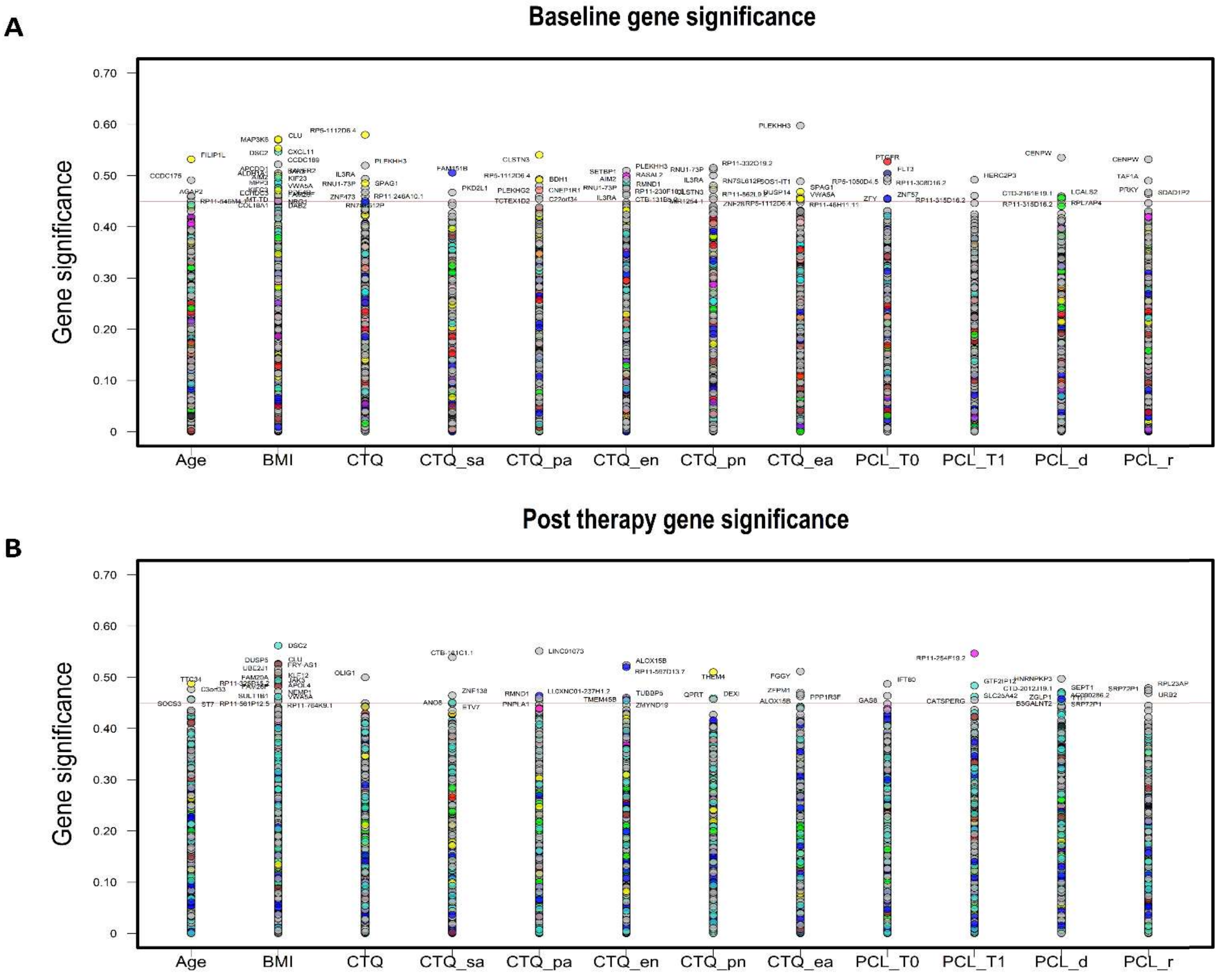

Figure A.4: Baseline and post therapy gene significance

Each point reflects a transcripts absolute correlation with the respective trait of interest (x-axis categories) before (A) and after application of psychotherapy (B). Colors give the module the transcript was assigned to during WGCNA analyses. Gene names are given for correlations surpassing a cutoff of *r* = .45.

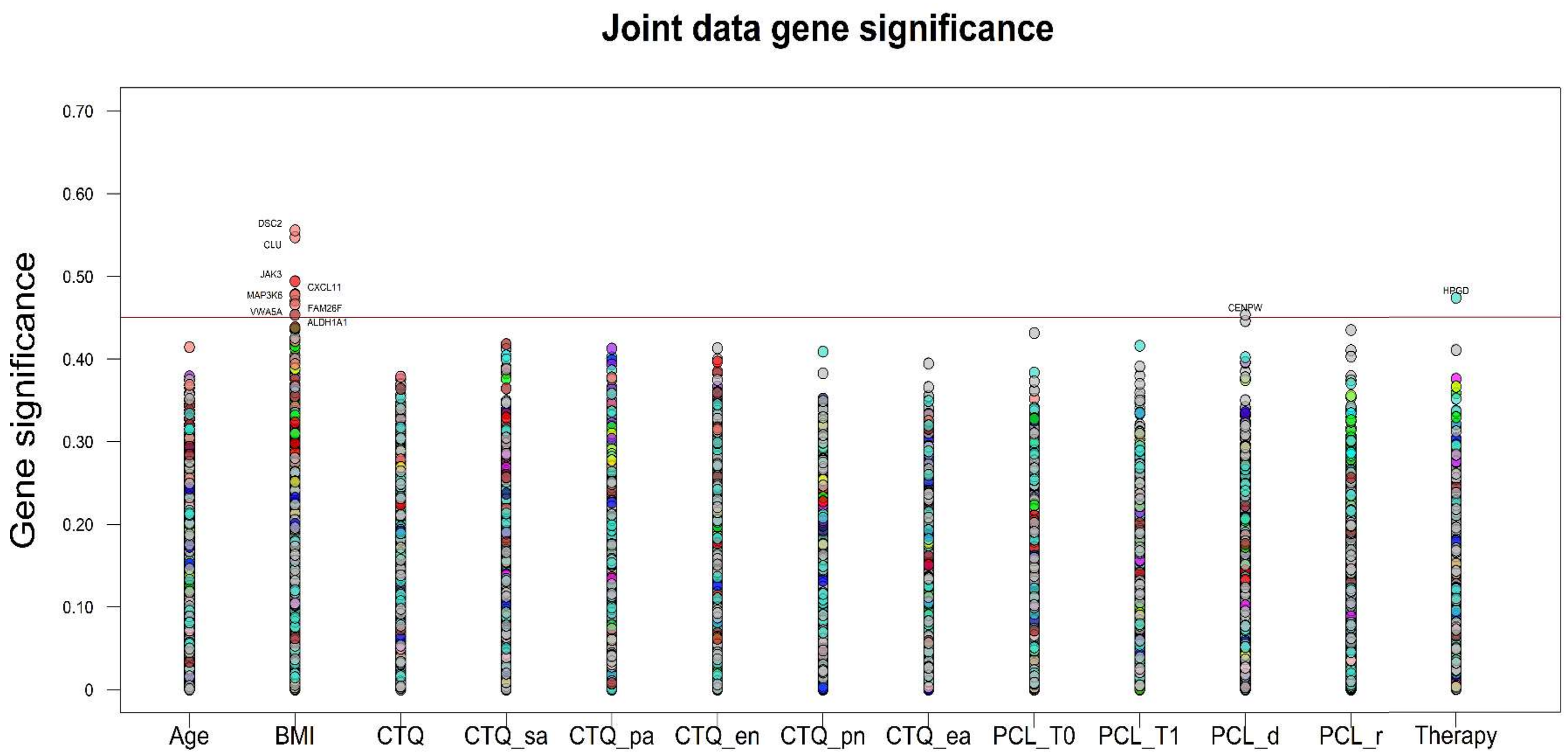

Figure A.5: Joint data gene significance

Each point reflects a transcripts absolute correlation with the respective trait of interest (x-axis categories). Colors give the module the transcript was assigned to during the WGCNA analysis. Gene names are given for correlations surpassing a cutoff of r = .45.

Table A.1: Module size and overlap with differentially expressed genes and trauma associated genes for all co-expression networks

**A**	**Baseline co-expression network**			
	module	size	DEG	TAG
1	black	114	-	11
2	blue	234	-	16
3	brown	163	-	10
4	cyan	32	-	1
5	green	150	2	16
6	greenyellow	40	1	2
7	magenta	69	3	25
8	midnightblue	32	4	7
9	pink	70	-	6
10	purple	60	-	10
11	red	122	2	15
12	salmon	36	1	9
13	tan	38	2	1
14	turquoise	252	3	71
15	yellow	162	14	44
	all	1574	32	244
	grey	3426	30	415
	∑	5000	64	659

**B**	**Post therapy co-expression network**			
	module	size	DEG	TAG
1	black	60	1	9
2	blue	430	4	27
3	brown	208	2	71
4	green	162	2	17
5	magenta	49	7	16
6	pink	53	1	3
7	red	78	-	8
8	turquoise	950	9	139
9	yellow	196	-	10
	all	2186	26	300
	grey	2814	36	359
	∑	5000	64	659

**C**	**Joint data co-expression network**			
	module	size	DEG	TAG
1	black	64	1	8
2	blue	286	1	33
3	brown	205	3	76
4	cyan	29	1	7
5	green	101	7	31
6	greenyellow	37	-	3
7	magenta	47	2	6
8	midnightblue	26	-	3
9	pink	49	-	11
10	purple	43	8	17
11	red	70	-	11
12	salmon	30	5	12
13	tan	32	-	-
14	turquoise	1199	21	84
15	yellow	152	1	17
	all	2370	50	319
	grey	2630	12	340
	∑	5000	64	659

DEG = differentially expressed genes after therapy, TAG = Trauma associated genes. Note: grey gives the default category, i.e. genes that were not assigned to a module.

Table A.2: Top 20 transcripts with highest kME values for all baseline modules. Genes are ranked based on their correlation in decreasing order First entries are likely to represent relevant hub genes. Please note that displayed genes are conserved about baseline and post-therapy network structures.

black	blue	brown	cyan	green	greenyellow	magenta	midnight blue	pink	purple	red	salmon	tan	turquoise	yellow
RNA5-8SP6	HSPE1P18	RP11-760D2.12	PFN1P1	PFN1P1	RNU6-951P	DHRS9	MME	RPS26P11	RNU6-758P	SEPT14	BTF3P4	RNU4ATAC	IFI44L	AHRR
RNU2-46P	RP11-310J24.3	NLRP9P1	RP11-34P13.15	TPT1P1	LARP4P	IL21R	RP11-536C10.1	AC004057.1	RP4-576H24.2	BX255923.2	CRTC2	MT-TC	LGALS3BP	LINC00599
RNU4ATAC	SNORA37	RP11-635N19.2	RPL13P2	RP4-604A21.1	SEPT14	RHOBTB3	MMP9	AC073072.7	Y_RNA	Y_RNA	RP11-423H2.1	MT-TY	IFI6	SEMA6B
RNU1-1	SMG1P5	YES1	U2AF1L4	TPT1P6	RN7SL481P	RUBCNL	CYP4F3	GS1-184P14.2	RPL12P13	RP11-10J21.2	GREM1	MT-TL2	IFIT1	TMEM45B
U2	AC005822.1	GCNT1P3	LAGE3P1	RPS27AP1	RP1-60N8.1	ADRB2	IL1R2	RPS26P8	SNORD3C	RBBP4P5	RP11-680H20.1	SNORD89	SERPING1	CTTNBP2
U2	RP11-325K4.2	RNU6-979P	TECRP1	RP11-422P24.9	NPIPB3	CD180	RP11-76E17.4	RP11-777J24.1	SNORD89	RP11-325K4.2	EEF1A1P12	MT-TH	EPSTI1	CLU
RNA5-8S5	PCBP2-OT1	CTA-276F8.1	TCEB2P2	RP11-343H5.4	RP11-456P18.2	C3AR1	ZBTB16	RP11-162A23.5	TMEM160	RP11-129H15.4	RP11-977G19.5	MT-TP	IFIT3	P2RY6
RPL21P111	Y_RNA	XXbac-BPG294E21.9	FKBP1C	RPL29P33	SNORA37	PRR5L	MGAM	RPS26P47	HIST2H2AC	Y_RNA	ZFP36	U2	IFIT2	PLIN5
SNORD3A	LRCH4	RP11-775J23.2	FTLP3	MIR1244-2	RP11-325K4.2	PTPRO	MMP25	AC008065.1	ANKRD10-IT1	CALM2P4	EEF1A1P29	SNORD13	LY6E	GPR183
RP11-230F18.6	RP11-73M18.7	RP11-484D2.4	GAPDHP63	RP11-680H20.1	PCBP2-OT1	DAB2	RN7SKP16	RPS26P28	EIF4BP3	RAP1BP3	TXNP6	NPIPB1P	NEXN	GPR82
U2	RNU1-124P	SNRPCP2	GTF2IP20	EEF1A1P12	Y_RNA	FFAR2	FLT3	RPS26P31	RN7SL587P	RNU1-124P	GRAMD4	MT-TN	IFI44	MMP25
FAM157C	MTRNR2L8	RPS27P23	MTX1P1	RP1-40G4P.1	RP11-166B2.1	TIFA	TFAMP1	RPS26P6	ADCK5	RP11-474D14.2	CXCL14	SMG1P5	RSAD2	MCTP2
RP11-325K4.2	HYMAI	RPL6P30	UBA52P5	EEF1A1P29	CCDC150P1	CX3CR1	AC012493.1	RP11-330L19.1	MMP17	NRBF2P3	AC009501.4	MT-TS2	GBP1	RGL1
PCBP2-OT1	DPRXP4	CTC-459F4.1	RP5-827C21.1	FTLP3	AJ003147.11	FCGR1CP	FAM101B	RPS26P15	WASH5P	MAK	CNEP1R1	MT-TV	GBP5	C1RL-AS1
AC097713.5	PBX2P1	OR52K2	GPX1P2	ANXA2P2	ADAMTSL4-AS1	FCGR1B	CXCR2	RPS26P13	GCHFR	ZNF638-IT1	HLA-F	MT-TE	OAS3	SASH1
RP11-166B2.1	NBPF26	ZNF273	VAMP2	RP11-142A22.3	DPRXP4	LAIR1	NAMPTP1	RPS26P35	DDX39B	MORF4	GTPBP2	MT-TR	HERC5	MAP3K6
MTRNR2L7	HNRNPKP2	PCBP2-OT1	RPLP0P6	RP11-829H16.2	NBPF26	ZNF703	SYCP2	RP11-713H12.1	HAS2	RP11-760D2.7	RPL23AP65	LINC00562	GBP1P1	MS4A4A
RP11-107F6.4	DDX39B	CTC-559E9.5	RP11-464D20.2	FTH1P8	SMG1P6	UBE2J1	GPR160	AC068522.4	SGTA	PBX2P1	TMEM128	MTND6P3	GBP4	CDKN1A
RP11-1094M14.1	SMG1P6	RP11-16E23.3	PAFAH1B3	EEF1A1P9	MTRNR2L12	FCGR1A	FKBP5	RPS26P3	MRPL28	NBPF26	TDRD7	C9orf47	SAMD9L	RUNX3
MTRNR2L12	MTRNR2L12	C9orf47	GPX1P1	RPL36AP26	RGPD2	CDK6	VNN3	NCF1B	DAPK3	HNRNPKP2	SDHD	MTRNR2L12	DDX60	FUCA1

Table A.3: Biotypes of Top 20 transcripts with highest kME values for all baseline modules. Genes are ranked based on their correlation in decreasing order First entries are likely to to represent relevant hub genes. Please note that displayed genes are conserved about baseline and post-therapy network structures.

black	blue	brown	cyan	green	greenyellow	magenta	midnight blue	pink	purple	red	salmon	tan	turquoise	yellow
rRNA	pr_ps	pr_ps	pr_ps	pr_ps	snRNA	pc	pc	pr_ps	snRNA	pc	pr_ps	snRNA	pc	pc
snRNA	sense_in	unpr_ps	pr_ps	pr_ps	tr_pr_ps	pc	pr_ps	tr_pr_ps	unpr_ps	unpr_ps	non_m_decay	Mt_tRNA	pc	lincRNA
snRNA	snoRNA	pr_ps	pr_ps	pr_ps	pc	pr_t	pc	pr_ps	misc_RNA	misc_RNA	pr_t	Mt_tRNA	pc	pc
snRNA	tr_unpr_ps	pc	non_m_decay	pr_ps	misc_RNA	pc	retained_i	pr_ps	unpr_ps	TEC	pc	Mt_tRNA	pc	pc
snRNA	pr_ps	pr_ps	pr_ps	pr_ps	sense_in	pc	pc	pr_ps	snoRNA	pr_ps	pr_ps	snoRNA	pc	pc
snRNA	sense_in	snRNA	pr_ps	pr_ps	pc	pc	lincRNA	pr_ps	snoRNA	sense_in	pr_ps	Mt_tRNA	pc	pc
rRNA	non_coding	lincRNA	pr_ps	pr_ps	pr_t	pc	pc	pr_ps	pc	pr_ps	sense_o	Mt_tRNA	pc	pc
pr_ps	misc_RNA	antisense	pc	pr_ps	snoRNA	pr_t	pc	pr_ps	pc	misc_RNA	pc	snRNA	pc	pc
snoRNA	pc	pr_ps	pr_ps	miRNA	sense_in	pc	pc	pr_ps	sense_in	pr_ps	pr_ps	snoRNA	pc	pc
sense_in	sense_in	sense_o	pr_ps	pr_ps	non_coding	pc	misc_RNA	pr_ps	pr_ps	pr_ps	pr_ps	pr_t	pc	pc
snRNA	snRNA	pr_ps	retained_i	pr_ps	misc_RNA	pr_t	pc	pr_ps	misc_RNA	snRNA	pc	Mt_tRNA	pr_t	pc
lincRNA	pc	pr_ps	unpr_ps	pr_ps	pc	pc	pr_ps	pr_ps	pr_t	pr_ps	pc	tr_unpr_ps	pr_t	pc
sense_in	non_coding	pr_ps	pr_ps	pr_ps	pr_ps	pc	pr_ps	pr_ps	pc	pr_ps	antisense	Mt_tRNA	pc	pc
non_coding	pr_ps	lincRNA	pr_ps	pr_ps	TEC	unpr_ps	pc	pr_ps	pr_t	pc	pc	Mt_tRNA	retained_i	antisense
pr_ps	pr_ps	pc	pr_ps	pr_ps	retained_i	retained_i	pc	pr_ps	pc	pr_t	pc	Mt_tRNA	pc	retained_i
pc	pc	pr_t	pc	TEC	pr_ps	pc	pr_ps	pr_ps	retained_i	pr_ps	non_m_decay	Mt_tRNA	pc	pc
pc	pr_ps	non_coding	pr_ps	pr_ps	pc	pc	pc	pr_ps	pc	pr_ps	pr_ps	lincRNA	pr_t	pc
pr_ps	retained_i	sense_in	pr_ps	pr_ps	unpr_ps	pc	pc	pr_ps	pc	pr_ps	pc	pr_ps	pc	pr_t
pr_ps	unpr_ps	sense_in	pc	pr_ps	pc	pc	pc	pr_ps	pc	pc	pc	pc	pc	pc
pc	pc	pc	pr_ps	pr_ps	pc	pc	pc	pr_t	pc	pr_ps	pc	pc	pc	pc

Ps = pseudogene, pc = protein coding, tr = transcribed, t = transcript, pr = processed, non = nonsense, in= intronic, i=intron, m = mediated, o = overlapping

Table A.4: Top 20 transcripts with highest kME values for all modules of the joint data co-expression network. Genes are ranked based on their correlation in decreasing order First entries are likely to represent relevant hub genes

black	blue	brown	cyan	green	greenyellow	magenta	midnightblue	pink	purple	red	salmon	tan	turquoise	yellow
RP11-78O7.2	SCARNA22	IFI44L	SYNE2	CA2	RPS26P11	LL22NC03-2H8.5	POU5F1B	RNU6-758P	AHRR	RPL13P2	FAM20A	CTB-25B13.5	RNU4ATAC	BTF3P4
THOC3	SNORD104	LGALS3BP	PGA3	EPS8	AC004057.1	ANP32BP3	RP11-214O14.1	DGKZP1	LINC00599	TECRP1	TMEM45B	MICALL2	SEPT14	RP4-604A21.1
PARP4P1	CRIP1P4	IFI6	MME	ADRB2	AC073072.7	RP11-366M4.2	RP4-604A21.1	Y_RNA	SEMA6B	RPL29P33	CLU	AC062017.1	HSPE1P18	TPT1P6
RPL30P3	SNORA12	IFIT1	CXCR1	CD180	GS1-184P14.2	PCP2	RPS27AP1	RPL12P13	TMEM45B	RP3-342P20.2	KIFC3	KCNMB3	SNORA37	CRTC2
RPL23AP55	TMEM191C	SERPING1	MMP9	AC093627.10	RPS26P8	HSP90AB2P	RP11-422P24.9	SNORD3C	CTTNBP2	FKBP1C	SLC39A8	Y_RNA	SMG1P5	RP11-422P24.9
AC104978.1	TUBG2	EPSTI1	DAPK2	PRR5L	RP11-777J24.1	RP11-70C1.1	CTD-2301A4.1	TMEM160	P2RY6	FTLP3	WASF1	CDC7	AC005822.1	MIR1244-2
AC021016.7	Y_RNA	IFIT3	CYP4F3	PTPRO	RP11-162A23.5	RP11-543E8.2	RP11-436H11.1	EIF4BP3	LINC01451	FIZ1	SOCS3	RPS27P23	RP11-325K4.2	RP11-680H20.1
GDF5OS	PPIAP11	IFIT2	MGAM	DAB2	RPS26P47	HMGB1P11	MT-ND1	RN7SL587P	PLIN5	GAPDHP63	MCTP2	RP11-345J4.4	PCBP2-OT1	EEF1A1P12
RP11-256K7.1	MTX1P1	LY6E	MMP25	TIFA	AC008065.1	RP11-618N24.1	FTLP3	ADCK5	GPR183	GTF2IP20	NRG1	CACNB3	Y_RNA	RP1-40G4P.1
TXNP6	EFEMP2	NEXN	FCGR3B	CX3CR1	RPS26P28	RP11-129H15.4	RPS4XP17	RETN	GPR82	AC012354.8	DAB2	RP11-499P20.2	LRCH4	RP11-977G19.5
RPL30P7	BCAT2	IFI44	PROK2	HDAC9	RPS26P31	HMGN5	MTND1P23	WDR24	FPR3	PPIAP11	C1RL-AS1	RPS26	RNU1-124P	BTF3P7
AC007283.4	RP4-761J14.10	RSAD2	LRRC4	FCGR1B	RPS26P6	ALS2CR12	RP11-142A22.3	MMP17	PRDM1	MTX1P1	DSC2	RN7SL612P	MTRNR2L8	EEF1A1P29
RP11-1094M14.1	GPX1P2	GBP1	SLCO4C1	HIVEP3	RP11-330L19.1	RNU6-1300P	CTD-2311M21.2	MVD	MMP25	UBA52P5	SASH1	SNORA77	HYMAI	FTLP3
RP11-485M7.1	RP11-175B9.2	GBP5	TNFRSF10C	LL21NC02-1C16.2	RPS26P15	RP11-430C1.2	MTND4P12	MTATP8P1	RGL1	RP5-827C21.1	MAP3K6	RP11-667M19.1	Y_RNA	CTD-2161E19.1
EEF1A1P22	DGKQ	OAS3	PPP1R3B	RHOBTB2	RPS26P13	LTB	MT-ATP8	GCHFR	GFRA2	RPS18P12	GPRIN3	POLR2J4	RGS17P1	ANXA2P2
AC009501.4	TMEM86B	HERC5	KCNJ15	LAIR1	RPS26P35	PSMC1P10	RPS18P12	MRPL23	SASH1	GPX1P2	MS4A4A	RP11-107F6.4	DPRXP4	FTH1P8
LLGL1	GPX1P1	GBP1P1	CXCR2	ZNF703	RP11-713H12.1	RP11-78H18.2	VAMP2	DYRK1B	LRG1	VAMP2	JAK3	RP4-548D19.3	NBPF26	EEF1A1P9
RPL23AP65	RP11-122G18.7	GBP4	CMTM2	UBE2J1	AC068522.4	RP11-760D2.7	PCIF1	SGTA	XYLT1	RPLP0P6	ADGRE1	RP11-215P8.3	DDX39B	FTH1P23
ATP5C1P1	RP11-251G23.2	SAMD9L	NAMPTP1	FCGR1A	RPS26P3	FCF1P8	C21orf33	MRPL28	RUNX3	PAFAH1B3	FLVCR2	CTD-2026D20.3	SMG1P6	RPL36AP26
CHCHD2P6	FAAH	DDX60	VNN2	CDK6	NCF1B	ARHGEF10L	RPL18AP15	DAPK3	FUCA1	GPX1P1	VWA5A	SNORA7	MTRNR2L12	RP11-270C12.3

Table A.5: Biotypes of the top 20 transcripts with highest kME values for all modules of the joint data co-expression network. Genes are ranked based on their correlation in decreasing order First entries are likely to represent relevant hub genes

**black**	**blue**	**brown**	**cyan**	**green**	**greenyellow**	**magenta**	**midnightblue**	**pink**	**purple**	**red**	**salmon**	**tan**	**turquoise**	**yellow**
lincRNA	scaRNA	pc	pc	pc	pr_ps	lincRNA	pc	snRNA	pc	pr_ps	pr_t	lincRNA	snRNA	pr_ps
pc	snoRNA	pc	retained_i	non_m_decay	tr_pr_ps	pr_ps	pr_ps	pr_ps	lincRNA	pr_ps	pc	retained_i	pc	pr_ps
unpr_ps	pr_ps	pc	pc	pc	pr_ps	pr_ps	pr_ps	misc_RNA	pc	pr_ps	pc	antisense	pr_ps	pr_ps
pr_ps	snoRNA	pc	pc	pc	pr_ps	pc	pr_ps	unpr_ps	pc	pr_ps	pr_t	pc	snoRNA	non_m_decay
pr_ps	non_m_decay	pc	pc	lincRNA	pr_ps	pr_t	pr_ps	snoRNA	pc	pc	pc	misc_RNA	tr_unpr_ps	pr_ps
pr_ps	pc	pc	non_m_decay	pr_t	pr_ps	antisense	pr_ps	pc	pc	pr_ps	pc	pc	pr_ps	miRNA
pr_ps	misc_RNA	pc	retained_i	pc	pr_ps	pr_ps	pr_ps	pr_ps	lincRNA	pc	pc	pr_ps	sense_in	pr_ps
pc	pr_ps	pc	pc	pc	pr_ps	pr_ps	pc	misc_RNA	pc	pr_ps	pc	TEC	non_coding	pr_ps
pr_ps	unpr_ps	pc	pc	pc	pr_ps	pr_ps	pr_ps	pr_t	pc	retained_i	pc	retained_i	misc_RNA	pr_ps
pr_ps	non_m_decay	pc	pc	pc	pr_ps	pr_ps	pr_ps	pc	pc	pr_ps	pc	antisense	pc	sense_o
pr_ps	pc	pr_t	pc	pc	pr_ps	pc	unpr_ps	pc	pc	pr_ps	antisense	pc	snRNA	pr_ps
pr_ps	lincRNA	pr_t	pc	retained_i	pr_ps	pc	TEC	pc	pc	unpr_ps	pc	misc_RNA	pc	pr_ps
pr_ps	pr_ps	pc	pc	pc	pr_ps	snRNA	pr_t	retained_i	pc	pr_ps	retained_i	snoRNA	non_coding	pr_ps
pr_ps	tr_pr_ps	retained_i	pc	antisense	pr_ps	pr_ps	pr_ps	unpr_ps	pc	pr_ps	pc	pr_ps	misc_RNA	pr_ps
pr_ps	pc	pc	pc	pc	pr_ps	pr_t	pc	pc	pc	pr_ps	pc	pr_t	pr_ps	pr_ps
antisense	retained_i	pc	pc	pc	pr_ps	pr_ps	pr_ps	pc	retained_i	pr_ps	pc	pr_ps	pr_ps	pr_ps
pc	pr_ps	pr_t	pc	pc	pr_ps	pr_ps	pc	pc	pc	pc	retained_i	antisense	pc	pr_ps
pr_ps	pr_ps	pc	pc	pc	pr_ps	pr_ps	pc	pc	pc	pr_ps	pc	antisense	retained_i	pr_ps
pr_ps	pr_ps	pc	pr_ps	pc	pr_ps	pr_ps	pc	pc	pc	pc	pc	pr_t	unpr_ps	pr_ps
pr_ps	pc	pc	non_m_decay	pc	pr_t	pc	pr_ps	pc	pc	pr_ps	pc	snoRNA	pc	pr_ps

ps = pseudogene, pc = protein coding, tr = transcribed, t = transcript, pr = processed, non = nonsense, in= intronic, i = intron, m = mediated, o = overlapping,

Table A.6: Enriched GO-Terms for genes upregulated following therapy

Term	P-value	Adjusted P-value	Genes
positive regulation of I-kappaB phosphorylation (GO:1903721)	8.06E-05	0.025085091	CX3CR1;TNF
negative regulation of cell differentiation (GO:0045596)	8.35E-05	0.025085091	SPRED1;CDK6;MYB;TNF;HDAC7
regulation of I-kappaB phosphorylation (GO:1903719)	1.13E-04	0.025085091	CX3CR1;TNF
regulation of smooth muscle cell proliferation (GO:0048660)	2.08E-04	0.034688543	TCF7L2;PTGIR;TNF
positive regulation of cell migration involved in sprouting angiogenesis (GO:0090050)	8.07E-04	0.066875415	HDAC9;HDAC7
leukocyte tethering or rolling (GO:0050901)	8.07E-04	0.066875415	CX3CR1;TNF
negative regulation of apoptotic signaling pathway (GO:2001234)	8.18E-04	0.066875415	CX3CR1;TCF7L2;TNF
regulation of NIK/NF-kappaB signaling (GO:1901222)	8.80E-04	0.066875415	TNFSF14;TNF;HDAC7
leukocyte adhesion to vascular endothelial cell (GO:0061756)	9.01E-04	0.066875415	CX3CR1;TNF
protein localization to membrane (GO:0072657)	0.001126215	0.073583821	LRRC4;ITGAL;TNF;SPTBN1
microglial cell activation (GO:0001774)	0.00121171	0.073583821	CX3CR1;TNF
inflammatory response (GO:0006954)	0.002062844	0.107011604	PTGIR;ITGAL;HDAC9;TNF
modulation of chemical synaptic transmission (GO:0050804)	0.002144737	0.107011604	CX3CR1;LRRC4;TNF
negative regulation of osteoblast differentiation (GO:0045668)	0.002406549	0.107011604	CDK6;HDAC7
tumor necrosis factor-mediated signaling pathway (GO:0033209)	0.002559885	0.107011604	TNFSF14;LTB;TNF
regulation of cell migration involved in sprouting angiogenesis (GO:0090049)	0.002563152	0.107011604	HDAC9;HDAC7
regulation of trans-synaptic signaling (GO:0099177)	0.003061	0.119647782	CX3CR1;LRRC4
regulation of apoptotic signaling pathway (GO:2001233)	0.003600445	0.119647782	CX3CR1;TNF
positive regulation of potassium ion transport (GO:0043268)	0.003600445	0.119647782	KCNMB1;KCNN4

Table A.7: Enriched GO-Terms for genes downregulated following therapy

Term	P-value	Adjusted P-value	Genes
adipose tissue development (GO:0060612)	2.43E-05	0.027149133	NAMPT;SH3PXD2B;ARID5B
cellular response to peptide hormone stimulus (GO:0071375)	1.75E-04	0.065729953	NR4A2;CYSLTR2;PDE3B;IRS2;CPEB2
cytokine-mediated signaling pathway (GO:0019221)	2.30E-04	0.065729953	ISG20;SOCS3;ANXA1;CEBPD;IL1R2;PELI1;IRS2;IL1RAP;HIF1A;EREG;MCL1
chronic inflammatory response (GO:0002544)	2.35E-04	0.065729953	VNN1;THBS1
regulation of long-chain fatty acid import across plasma membrane (GO:0010746)	3.52E-04	0.067663099	IRS2;THBS1
response to metal ion (GO:0010038)	4.10E-04	0.067663099	PLSCR1;AQP9;HIF1A;THBS1
cellular response to insulin stimulus (GO:0032869)	4.34E-04	0.067663099	NAMPT;PDE3B;PDK4;IRS2;CPEB2
regulation of oxidative stress-induced neuron intrinsic apoptotic signaling pathway (GO:1903376)	4.91E-04	0.067663099	HIF1A;MCL1
cellular response to cytokine stimulus (GO:0071345)	6.01E-04	0.067663099	SOCS3;ANXA1;CEBPD;DUSP1;IL1R2;IL1RAP;HIF1A;EREG;MCL1
negative regulation of phospholipase activity (GO:0010519)	6.53E-04	0.067663099	RGS2;ANXA1
cellular response to hydrogen peroxide (GO:0070301)	6.66E-04	0.067663099	OSER1;ANXA1;ECT2
connective tissue development (GO:0061448)	8.50E-04	0.07397052	NAMPT;SH3PXD2B;ARID5B
response to cytokine (GO:0034097)	8.61E-04	0.07397052	PLSCR1;ANXA1;KYNU;CXCL16;MCL1
negative regulation of transmembrane transport (GO:0034763)	0.001042412	0.083169583	IRS2;THBS1
T cell chemotaxis (GO:0010818)	0.001269995	0.094572305	GPR183;CXCL16
regulation of B cell proliferation (GO:0030888)	0.001486387	0.099691693	GPR183;IRS2;AHR
negative regulation of cardiac muscle hypertrophy (GO:0010614)	0.001519135	0.099691693	RGS2;LMNA
positive regulation of blood vessel endothelial cell migration (GO:0043536)	0.001681641	0.099691693	ANXA1;HIF1A;THBS1
regulation of smooth muscle cell proliferation (GO:0048660)	0.00178499	0.099691693	TRIB1;THBS1;EREG
